# Synergistic TCR-independent action of IL-33 and IL-12 drive a potent IFNγ secretory program in human circulating MAIT cells with immunomodulatory properties

**DOI:** 10.3389/fimmu.2026.1852383

**Published:** 2026-06-26

**Authors:** Carlota García-Escribano, Maria Gallardo-Jiménez, Ana García-Cadarso, Paloma Fernández Martínez, Ricardo Arroyo-Solera, Luis Senador Zaldívar-Martínez, Kelin Lin, Jeffrey Aubé, Patricia Barral, Tomás Chivato, Domingo Barber, Maria M. Escribese, Elena Izquierdo, Juan Carlos López-Rodríguez

**Affiliations:** 1 Departamento de Ciencias Médicas Básicas, Facultad de Medicina, Instituto de Medicina Molecular Aplicada-Nemesio Díez (IMMA-ND), Universidad San Pablo-CEU, CEU Universities, Boadilla del Monte, Spain; 2 Division of Chemical Biology and Medicinal Chemistry, UNC Eshelman School of Pharmacy, University of North Carolina at Chapel Hill, Chapel Hill, NC, United States; 3 The Peter Gorer Department of Immunobiology, King’s College London, London, United Kingdom

**Keywords:** IFNγ, IL-12p70, IL-33, MAIT cells, P38-MAPK, secretome, Glycolysis, monocytes

## Abstract

**Introduction:**

Mucosal-Associated Invariant T (MAIT) cells are a subset of unconventional T cells that rapidly respond to early signs of inflammation, infection, and tissue damage. While MAIT cells have been typically associated with microbial infections, given their ability to respond to both microbial-derived riboflavin metabolites and proinflammatory cytokines such as IL-12, IL-15, or IL-18, their role in other inflammatory conditions remains mostly unknown. Alarmins, including IL-25, IL-33, and thymic stromal lymphopoietin (TSLP), are crucial effectors for early inflammatory responses, being released upon epithelial damage and strongly promoting the polarization of a wide variety of immune cells, including leukocytes like neutrophils, monocytes, macrophages, dendritic cells (DCs), NK cells, ILCs and T cells. However, how alarmins-induced environments influence MAIT cells' activity and function is yet to be explored.

**Methods:**

In this study, we investigate the roles of alarmins in controlling MAIT cell activation and function.

**Results:**

We found that IL-33, but not IL-25 or TSLP, combined with the TCR-independent stimuli IL-12p70 (but not 5-OP-RU), induces a potent IFNγ secretory/cytotoxic program on MAIT cells. This response was strongly dependent on p38 MAPK signaling and glycolytic metabolism. Beyond IFNγ, IL-33/IL-12p70-activated MAIT cells secrete a diverse panel of immune mediators, including TNF, VEGF, OSM, CXCL11, and CCL3. Notably, conditioned media from purified Vα7.2^+^ T cells (enriched on MAIT cells) were able to polarize CD14^+^ monocytes towards a M1-like inflammatory phenotype, increasing both the expression of proinflammatory genes and phagocytic capacity.

**Discussion:**

These findings reveal a broader immunomodulatory potential for MAIT cells to influence diverse immune compartments during inflammatory response.

## 1 Introduction

Unconventional T cells represent a subset of T lymphocytes that bridge innate and adaptative immune response, as they exhibit characteristics from both compartments such as rapid activation and the ability to recognize microbial derived antigens through their T Cell Receptors (TCR) (1). Among unconventional T cells, Mucosal-Associated Invariant T (MAIT) cells have awakened interest in recent years given their potential to produce a broad range of immunomodulating molecules that take part in many inflammatory situations, ranging from microbial infections to tissue repair (2).

MAIT cells contribute to early immune responses against bacterial and fungal infections recognizing metabolites derived from riboflavin metabolism via MR1-dependent antigen presentation. In humans, the majority of MAIT cells are defined as CD8^+^CD161^+^Vα7.2^+^ T cells, as their semi-invariant TCR typically comprises a concrete TCRα chain (encoded by *TRAV1-2/TRAJ33* genes) that pairs with a limited repertoire of TCRβ chains (TRAJ33/20/12) (3–5). Moreover, they contribute to the response against viruses predominantly through antigen-independent, cytokine-driven (e.g., IL-12, IL-15, IL-18), activation (6). Once activated, MAIT cells rapidly produce high levels of proinflammatory cytokines including IFNγ, TNF, and IL-17, as well as other immune mediators that promote cytotoxicity (e.g. Granzyme B, perforin) or tissue repairing (e.g. TGFβ, VEGF, IL-10), depending on the inflammatory context (7). Despite increasing evidence for cytokine-mediated MAIT activation, the contribution of other early immune mediators, such as alarmins, remains largely unexplored.

Alarmins, including IL-25, IL-33, and thymic stromal lymphopoietin (TSLP), are a set of proinflammatory molecules rapidly released upon epithelial damage serving as early activation signals that alert the immune system to tissue stress or injury (8, 9). They have been described to influence both innate and adaptative immune cells, triggering activation in dendritic cells (10), some ILC subsets (e.g. ILC2s) (11) and CD4^+^ T cells (12, 13), and other unconventional T cells like invariant Natural Killer T Cells (iNKTs) (14–16). However, the possible effects of alarmins in other less explored immune cells involved in early inflammatory responses, such as MAIT cells, remain unknown.

Understanding how these early inflammatory mediators affect MAIT cells would help further clarify process controlling MAIT cell functional polarization (towards MAIT1, secreting IFNγ and/or cytolytic agents, or MAIT17 subsets, secreting IL17A), activation and function in non-microbial inflammatory conditions such as Type 2 inflammation, airway remodeling or autoimmune processes. Hence, this study aims to evaluate how alarmins, alone or in combination with established MAIT cell-related stimuli (TCR-dependent such as the riboflavin derivative 5-OP-RU or cytokines like IL-12p70), affect MAIT cell activation and effector functions; and explore their potential immunomodulatory consequences.

## 2 Methods

### 2.1 Isolation of human PBMCs

PBMCs from healthy donors were isolated from buffy coats kindly collected and donated for research purposes by the Centro de Transfusiones de la Comunidad de Madrid (Community of Madrid Transfusion Centre, Spain). PBMCs were obtained through Ficoll^®^ density gradient centrifugation and treated with Red Blood Cells Lysis Buffer eBioscience™ (Invitrogen™) before being frozen in FBS (Gibco) 10% DMSO (Sigma-Aldrich^®^) until its use.

### 2.2 *In vitro* cell culture – MAIT cells

In humans, circulating MAIT cells are mostly (>80% are CD8^+^) defined as Vα7.2^+^ CD161^+^ T cells (17). For this study we established three different culture approaches to study MAIT cell biology. MAIT cells were evaluated among whole PBMCs (representing 0.01-5% of total PBMCs) (**Supplementary Figure 1A**), purified Vα7.2^+^ T cells (from which MAITs represent up to 50-70% of total) (**Supplementary Figure 1B**) and purified MAIT cells using a 5-OP-RU-loaded MR1 tetramer (**Supplementary Figure 1C**).

For whole PBMCs cultures, 1x10^6^ cells were seeded in 96 Well Round (U) Bottom Plate (Grynia) for 48 h in complete RPMI medium (RPMI 1640 medium with GlutaMAX™ (Gibco)), 10% FBS, Penicillin-Streptomycin 100 U/mL (Gibco), Sodium Pyruvate 1 mM (Gibco), Non-Essential Amino Acids (Gibco), 0.1% β-mercaptoethanol (Sigma-Aldrich^®^)). Cells were cultured for 48 hours in the presence of Recombinant Human IL-2 (Biolegend) (10 ng/mL), Recombinant Human IL-7 (Biolegend) (10 ng/mL) and stimulated with combinations of the following reagents: Recombinant Human IL-12p70 (Biolegend) (50 ng/mL), Recombinant Human IL-33 (Biolegend) (50 ng/mL), Recombinant Human IL-25 (Biolegend) (50 ng/mL), Recombinant Human TSLP (Biolegend) (50 ng/mL), and 5-OP-RU (0.5 mM), which was prepared by mixing 5-A-RU (1 mM) and methylglyoxal (Sigma-Aldrich^®^) (50 mM). Cells were incubated at 37 °C, 90% humidity with 5% CO_2_.

For Vα7.2^+^ T cell cultures (enriched on MAIT cells), cells were isolated from PBMCs using a combination of PE-conjugated αVα7.2 (Biolegend) and αPE microbeads (Miltenyi Biotec). Cells were purified using LS columns (Miltenyi Biotec) and QuadroMACS™ Separator magnets (Miltenyi Biotec), following manufacturer’s instructions. 2x10^5^ Vα7.2^+^ cells were then cultured in 96 Well Round (U) Bottom Plate for 48 hours in RPMI medium in the presence of IL-2 (10 ng/mL), IL-7 (10 ng/mL) and combinations of the following reagents: IL-12p70 (50 ng/mL), IL-33 (50 ng/mL), p38 MAPK specific inhibitor (VX-745, 5 µM, Sigma-Aldrich^®^) (18), STAT4 specific inhibitor (Lisofylline, 20 μM, Sigma-Aldrich^®^) (19), glycolysis inhibitor (2-Deoxy-D-glucose (2-DG), 1 mM, Sigma-Aldrich^®^) (20) and OXPHOS inhibitor (Oligomycin, 2 µM, Sigma-Aldrich^®^) (21). Condition media from Caco-2 cells or monocyte-derived dendritic cells (moDCs) were added at 1:5 dilution. 

### 2.3 *In vitro* cell culture – CD14^+^ monocytes, moDCs and Caco-2 cells

For CD14^+^ monocyte cultures, cells were isolated from whole PBMCs using an anti-CD14 antibody (Miltenyi Biotec) as described before. 2x10^5^ CD14^+^ cells were seeded in 96 Well Flat Bottom Plate (Grynia) for 24 h in complete RPMI medium in the presence of the following treatments: Recombinant Human IFNγ (Biolegend) (100 ng/mL), Recombinant Human IL-33 (50 ng/mL) + Recombinant Human IL-12p70 (50 ng/mL), media derived from culturing Vα7.2^+^ T cells in control conditions (1:2 dilution in complete RPMI) or media derived from culturing Vα7.2^+^ T cells in presence of IL-12p70+IL-33 (1:2 dilution in complete RPMI) as explained before. In order to discard effects coming from the residual cytokine carryover, a Vα7.2^+^ T cell-free control media including only IL-12p70 and IL-33 (IL-12 + IL-33) were also included in the analysis. After 24 h cells were lysed for RNA extraction or processed for phagocytosis assays.

Monocyte-derived dendritic cells (moDCs) were differentiated from CD14^+^ cells. Briefly, 2x10^5^ CD14^+^ cells were seeded in 96 Well Flat Bottom Plate in RPMI medium in the presence of Recombinant Human GM-CSF (Biolegend) (70 ng/mL) and Recombinant Human IL-4 (Biolegend) (50 ng/mL), following a differentiation protocol previously described (22). After differentiation, moDCs were treated with LPS from E. coli O111:B4 (Sigma-Aldrich^®^) (250 ng/mL) for 24 h. Culture media was collected for subsequent Vα7.2^+^ T cell culture experiments.

Caco-2 human colon cancer cell line (ATCC HTB-37) were maintained in DMEM media (Gibco) supplemented with Penicillin-Streptomycin 100 U/mL and 10% FBS. Cells were seeded in a P12 Well flat Bottom Plate (Grynia) and once 75% cell confluency was reached, treated with LPS (2 μg/mL) for 24h. Culture media was collected for Vα7.2^+^ T cell culture experiments.

After the indicated culture hours, culture media was collected for ELISA assays, and cells were either processed for flow cytometry or stored in RNeasy Lysis (RLT) buffer containing 1% *β*-mercaptoethanol at -80 °C for subsequent RNA extraction and RT-qPCR analysis.

### 2.4 Flow cytometry

For experiments including intracellular staining, cells were treated with Brefeldin A Solution (Invitrogen) for 3 hours before starting flow cytometry staining. Staining was performed in FACs buffer (5% FBS, 0.02% sodium azide (Sigma-Aldrich^®^)) using anti-human antibodies from Biolegend (Detailed in **Supplementary Table 1**) at a final concentration of 1:200 for surface antibodies and 1:100 for intracellular antibodies. Dead cells were excluded using LIVE/DEAD Viability/Cytotoxicity Kit (Thermofisher Scientific) and Fc receptors were blocked with Human TruStain FcX™ (Biolegend) prior to surface marker staining. For intracellular staining, cells were fixed with Cyto-Fast™ Fix/Perm Buffer Set (Biolegend). For viability assays, cells were stained with annexin V after surface marker staining, distinguishing between Live Cells (Zombie^−^ Annexin V^+^), apoptotic cells (Zombie^−^ Annexin V^+^), necrotic cells (Zombie^+^ Annexin V^−^), and necro-apoptotic cells (Zombie^+^ Annexin V^+^). As apoptosis control, cells were treated for 6 h prior to the staining protocol with Staurosporine, *Streptomyces sp* 1 µM (Sigma-Aldrich^®^) (23). For CD64 surface staining, samples were prepared following the flow cytometry protocol explained before. CD14^+^ monocytes were stained using an anti-CD64 (**Supplementary Table 1**). Lastly, samples were analyzed using Attune NxT Flow Cytometer (Thermofisher Scientific) and data was processed using FlowJo software (TreeStar). Human MAIT cells were gated as Zombie^−^CD14^−^CD19^−^CD8α^+^CD161^+^Vα7.2^+^ cells as described before (24).

### 2.5 Phagocytosis assays

Phagocytosis was also evaluated using pHrodo™ Green BioParticles™ (Invitrogen), following manufacturer’s instructions. Briefly, CD14^+^ Monocytes were collected after culture under the conditions previously detailed with Vα7.2^+^ T cell-derived media and incubated for 30 minutes with pHrodo BioParticles at a final concentration of 1:10 at 37 °C. After the incubation, cells were washed and treated for 15 minutes with PBS-PFA 2%. Lastly, cells were washed again, resuspended in FACs buffer and analyzed as previously described.

### 2.6 ELISA

Medium supernatants were collected after culture. ELISA was performed in 96-well EIA/RIA Clear Flat Bottom Polystyrene High Bind Microplate (Corning) using ELISA MAX™ Standard Set Human IFN-γ, IL-10 and TNFα (Biolegend) following manufacturer’s instructions. Optimized amount of culture supernatants was used respectively for each ELISA assay. The plate was read at 450 nm with a Varioskan LUX Multimode Microplate Reader (Thermo Scientific).

### 2.7 RT-qPCR

RNA was purified using RNeasy Mini Kit (Qiagen) following the manufacturer’s instructions. During the extraction, samples were treated with RNase-Free DNase Set (Qiagen). RNA concentration was measured using NanoDrop™ 2000/2000c (Thermo Fisher Scientific) and then retrotranscribed to cDNA with the High-Capacity RNA-to-cDNA™ Kit (Applied Biosystems) in a Mastercycler gradient Thermal Cycler (Eppendorf).

RT-qPCR was performed using TB Green Premix Ex Taq (Takara) in a QuantStudio™ 5 Real-Time PCR System 384-well (Thermo Fisher Scientific). Sequences for all the primers used can be found in **Supplementary Table 2**. For qPCR analysis, the FC (Fold Change) was calculated as 2^-ΔΔCt^ (25), using gene *18S* as the housekeeping gene.

### 2.8 MAIT cell-derived secretome analysis

MAIT cells were isolated from PBMCs using a 5-OP-RU-loaded PE-conjugated MR1 tetramer (kindly provided by the National Institutes of Health (NIH) Tetramer Facility) combined with αPE microbeads/QuadroMACS™ Separator magnets (Miltenyi Biotec), as previously reported (26). 2x10^5^ MAITs were then cultured in a 96 well round (U) bottom plate for 48 hours in the presence of IL-2 (10 ng/mL), IL-7 (10 ng/mL) and combinations of the following reagents: IL-12p70 (50 ng/mL), IL-33 (50 ng/mL).

Proteomic characterization of the secretome was performed using media derived from culturing these tetramer-enriched MAIT cells (conditions: un-stimulated, IL-33, IL-12p70 and IL-33+IL-12p70) by Proximity Extension Assay (PEA; Olink^®^, Sweden). The samples were analyzed with the Olink^®^ Target 48 Cytokine panel including 40 proteins. All samples were revised for quality control, and protein levels are reported as absolute concentration values (pg/mL). In order to standardize data analysis, z-score for each protein concentration (pg/mL) was calculated according to the formula ‘z=(x - μ)/σ’, where ‘x’ is the concentration of the protein (pg/mL), ‘μ’ is the population mean for each protein, and ‘σ’ is the population standard deviation for each protein.

### 2.9 Statistical analysis

All statistical analyses were performed in Prism GraphPad Software (version 8.0.1). The normality of the groups was analyzed using Shapiro-Wilk tests and comparisons between groups were performed using Student’s t-tests, Mann–Whitney U test or one-way ANOVA. The mean ± SEM is displayed in each data set.

## 3 Results

### 3.1 Alarmins and IL-12p70 induced a potent IFNγ secretory program in circulating MAIT cells

To begin assessing the role of MAIT cells in response to alarmins, PBMCs were stimulated with the alarmins (IL-25, IL-33 and TSLP) in combination with 5-OP-RU and/or IL-12p70.

We identified the MAIT cell compartment by flow cytometry among whole PBMCs as CD8^+^CD161^+^Vα7.2^+^ T cells (**Supplementary Figure 1A**), as previously reported by Koay H et al. (27), and measured MAIT cell activation and effector functions by quantifying surface marker expression and intracellular cytokines. MAIT cell frequency remained unchanged across all conditions tested (**Supplementary Figure 2A**), and no significant differences in cell viability were detected, as assessed by Zombie/Annexin V staining (**Supplementary Figure 2B**).

**Figure 1:**
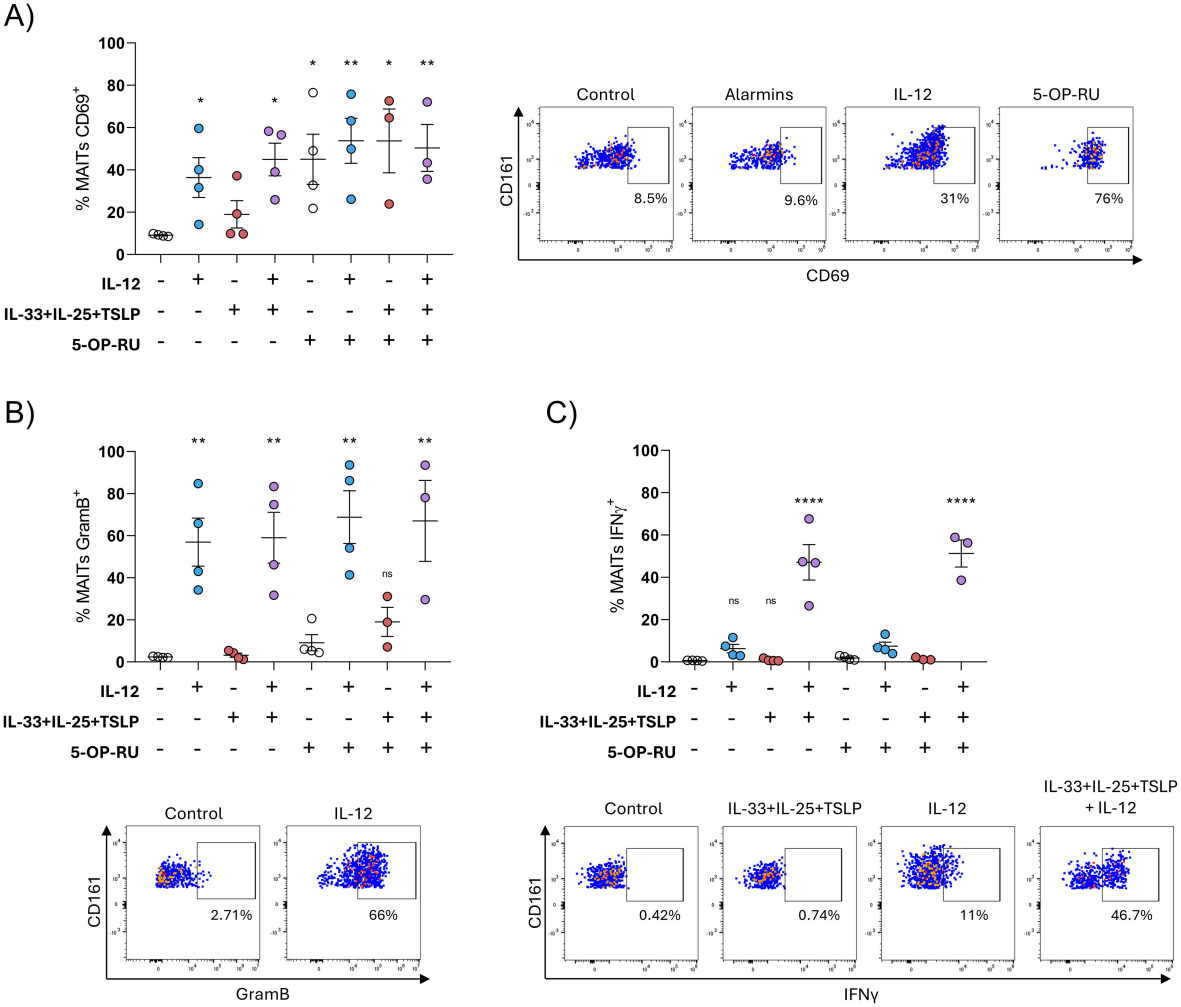
Alarmins and IL-12p70 induce an IFNγ secretory program on circulating MAITs. Whole PBMCs were stimulated with IL-33, TSLP and IL-25 in the presence of MAIT TCR-independent (IL-12p70) and -dependent (5-OP-RU) stimuli for 48h before flow cytometry analysis. **(A–C)** Bar charts and representative flow cytometry plots showing changes in MAIT cell alterations after indicated treatments in **(A)** activation (CD69+), **(B–C)** function (GramB+, IFNγ+). Bars represent Mean ± SEM, each dot is an independent donor (n = 3-4). ns, not significant; *P < 0.05, **P < 0.01, ****P < 0.0001 ANOVA with multiple comparisons test.

MAIT cell activation was measured by mean of CD69 expression. Our results show that, while IL-12p70 and/or 5-OP-RU triggered a significant activation of MAIT cells (mean: 40-50% CD69^+^ of total cells), the alarmins themselves could not induce a significant activation of circulating MAIT cells (vs unstimulated MAITs) (**Figure 1A**). Furthermore, co-stimulation of IL-12p70 and/or 5-OP-RU with the alarmins did not further increase the frequency of CD69^+^ MAIT cells.

Next, we evaluated if alarmins could alter MAIT cell effector function (**Figures 1B, C**). We observed that neither alarmins nor 5-OP-RU induced the secretion of Granzyme B, being exclusively elevated by the presence of IL-12p70 (reaching ~60% of total MAIT cell population) (**Figure 1B**). However, while neither alarmins nor 5-OP-RU alone did alter IFNγ secretion, presence of alarmins potentiated IL-12p70-induced IFNγ production, increasing the frequency of IFNγ^+^ MAITs from ~5% to 40% (**Figure 1C**). Stimulation with 5-OP-RU did not further modify this IFNγ induction. Of note, the percentage of double positive MAIT cells for IFNγ and Granzyme B was also analyzed (**Supplementary Figure 2C**), finding that IFNγ-producing cells induced by the combination of alarmins and IL-12p70 also exhibited cytotoxic capacities.

Lastly, to evaluate if the synergistic effect of alarmins and IL-12p70 is specific to the MAIT cell compartment, we evaluated the Granzyme B and IFNγ production in non-MAITs CD8^+^ T cells (defined as CD8^+^ T cell fraction but excluding CD161^+^ Vα7.2^+^ cells, **Supplementary Figure 2D**) under the same stimulatory conditions. We found that alarmins+IL-12p70 also induced Granzyme B and IFNγ production by non-MAIT CD8^+^ T cells, although to a lesser extent than in MAIT cells.

### 3.2 IL-33, in combination with IL-12p70, is responsible for the robust IFNγ production on circulating MAIT cells

**Figure 2:**
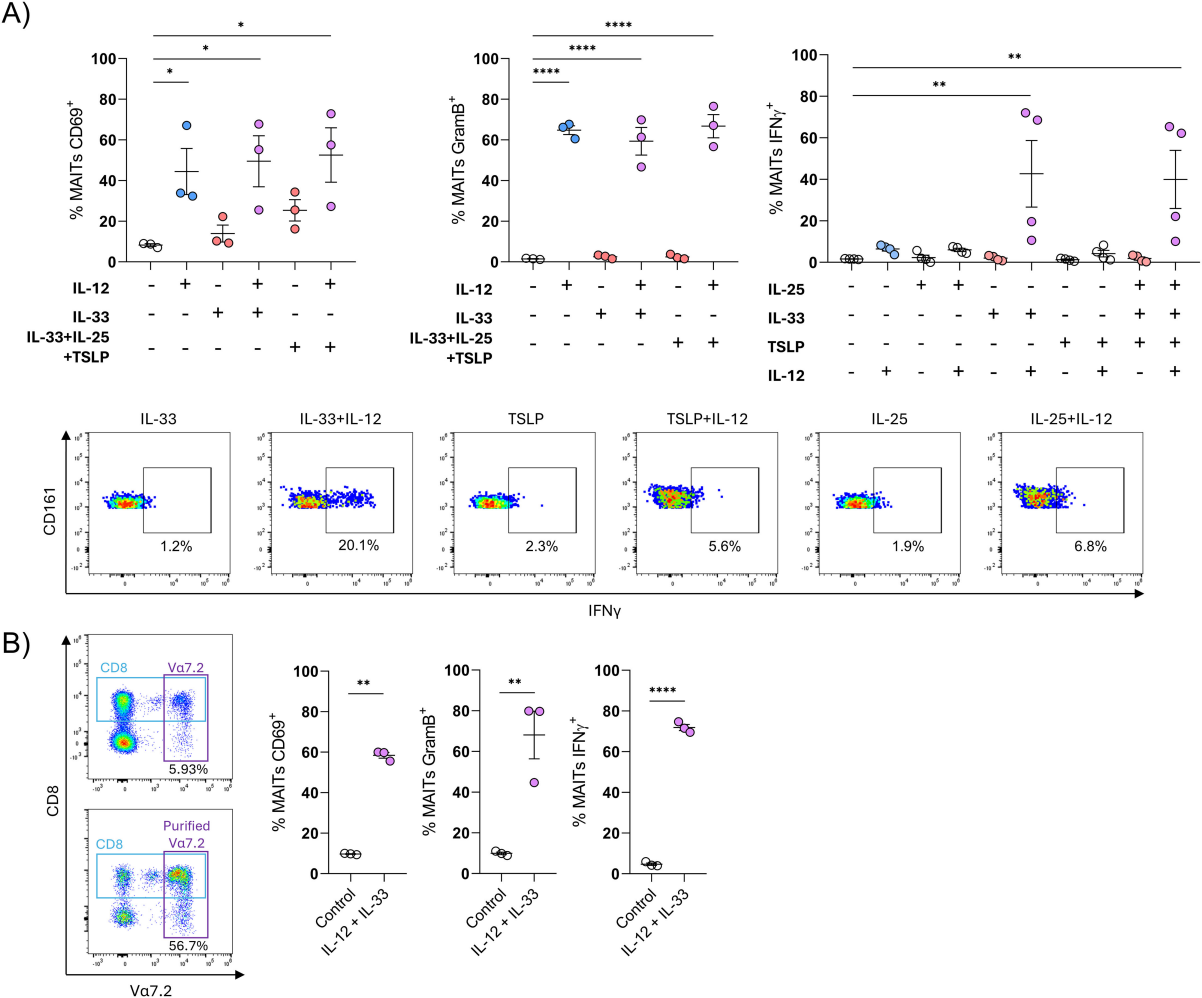
IL-33 and IL-12p70 are behind the potent IFNg-secretory program induced on human circulating MAITs. **(A)** Whole PBMCs were cultured for 48h in the presence of IL-33, IL-25, TSLP or the three alarmins together (IL-33+IL-25+TSLP) and/or in combination with IL-12p70. The figure includes bar charts (top) and representative flow cytometry plots (bottom) for the percentage of MAITs CD69+, GramB+, and IFNγ+. (B) Vα7.2+ cells (enriched on MAIT cells) were purified and cultured for 48h in the presence of IL-33 and IL-12p70 (left flow cytometry plots). Bars represent mean ± SEM, each dot is an independent donor (n = 3). ns, not significant; *P < 0.05, **P < 0.01, ****P < 0.0001 ANOVA with multiple comparisons test.

After confirming that IL-12p70 combined with alarmins induces robust IFNγ production in MAIT cells, we tested whether this effect required all three alarmins (IL-33, IL-25, TSLP). PBMCs were treated with each alarmin alone or with IL-12p70, and MAIT cells were analyzed for activation (CD69), Granzyme B, and IFNγ (**Figure 2A**, **Supplementary Figure 3A**). Only IL-12p70 with IL-33, but not IL-25 or TSLP, significantly increased IFNγ secretion (~10-fold vs unstimulated), and these IFNγ^+^ MAIT cells also expressed Granzyme B, indicating cytotoxic potential (**Supplementary Figure 3B**). We also evaluated the expression of *TBX21* and *RORGT* (**Supplementary Figure 3C**), transcription factors regulating type 1 (MAIT1) and type 17 (MAIT17) differentiation programs (28–30) in purified Vα7.2^+^ T cells (where MAIT cells represent ~50% of all of them, **Supplementary Figure 1B**), respectively. Our results show a significant upregulation of *TBX21* and a concomitant downregulation of *RORGT* after treatment with IL-12p70 and IL-33. Together with the potent IFNγ secretion, these findings indicate a polarization of circulating MAIT cells towards a MAIT1 phenotype. To assess additional effects, exhaustion (PD-1) and proliferation (Ki-67) markers were measured (**Supplementary Figure 3D**). IL-12p70 increased PD-1 expression, while alarmins had no further impact; Ki-67 levels remained unchanged.

To determine whether IL-33/IL-12p70 acted directly on MAIT cells, purified Vα7.2^+^ T cells (enriched on MAIT cells) were stimulated in the absence of other PBMCs (**Figure 2B**). IL-33/IL-12p70 stimulation induced MAIT (CD8^+^CD161^+^Vα7.2^+^) cell activation (CD69 expression), Granzyme B and IFNγ production. In addition, this combined treatment upregulated *IL12RB2* (which encodes for IL-12p70 receptor subunit), and specifically increased *ST2* (which encodes for IL-33 receptor) in response to IL-33 (**Supplementary Figure 3E**), confirming that MAIT cells (purified Vα7.2^+^ T cells) express both receptors and respond independently of other cell types.

Finally, to prove that MAIT cells can respond to endogenously produced IL-33 and/or IL-12p70, we used conditioned media from two different cellular systems: Caco-2 cells and moDCs, which release IL-33 or IL-12p70, respectively, upon LPS stimulation (**Supplementary Figure 4**). Caco-2 cells have been previously described to produce IL-33 in response to LPS stimulation (31). Similarly, moDCs have been reported to be an important source of IL-12p70 after LPS-recognition (32). Media derived from LPS-exposed moDCs or Caco-2 cells significantly increased IFNγ production in MAIT (purified Vα7.2^+^ T cells) cells when combined with IL-33 or IL-12p70, respectively, supporting the results obtained with recombinant cytokines (**Supplementary Figure 4**).

Collectively, our results point out that IL-33 directly enhances IL-12p70-driven IFNγ production in circulating MAIT cells.

### 3.3 IL-33/IL-12p70-induced IFNγ secretion on circulating MAIT cells is mechanistically controlled by p38-MAPK signaling and relies on glycolysis

**Figure 3:**
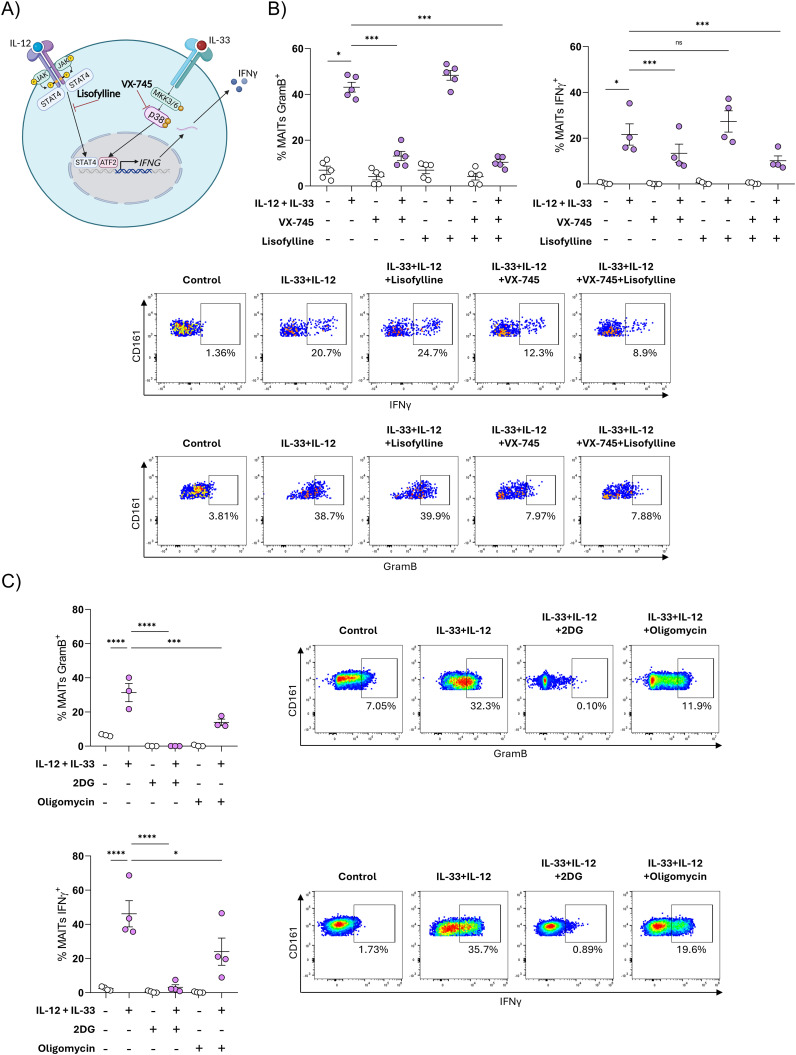
Molecular mechanisms controlling the IFNg-secretory program induced by IL-33/IL-12p70 on human circulating MAITs. **(A)** Graphic scheme of the inhibitors used and pathways affected. **(B)** Purified Vα7.2+ T cells were cultured for 48h in the presence of IL-33+IL-12p70, VX-745 (p38 inhibitor), and/or Lisofylline (STAT4 inhibitor). Bar charts (top) and representative flow cytometry plots (bottom) showing the percentage of GramB+ and IFNγ+ MAITs cells. **(C)** Purified Vα7.2+ T cells were cultured for 48h in presence of IL-33+IL-12p70, 2DG (glycolysis inhibitor) and/or Oligomycin (OXPHOS inhibitor) for 48h. The figure includes bar charts (left) and representative flow cytometry plots (right) showing the percentage of GramB+ and IFNγ+ MAITs cells. Bars represent mean ± SEM, each dot is an independent donor (n = 3-5). ns, not significant; *P < 0.05, ***P < 0.001, ****P < 0.001 Student’s t-test. Images contained in this figure were created with BioRender.com.

Our next objective was to determine the molecular mechanisms underlying IFNγ secretion by MAIT cells in response to IL-33/IL-12p70. In parallel, we also examined the pathways controlling cytotoxic capabilities, as measured by Granzyme B expression. Previous works in human CD8^+^ T cells point to a major dependence on p38 MAPK signaling and STAT4 activation upon exposure to IL-33 and IL-12p70, respectively (33). To assess if similar mechanisms operate in MAIT cells, whole PBMCs were treated with IL-33 and IL-12p70 in the presence of the p38-MAPK specific inhibitor VX-745 (18), the STAT4 specific inhibitor Lisofylline (19) or both (**Figure 3A**). Our results showed that, inhibition of p38-MAPK signaling significantly reduced both Granzyme B and IFNγ production in circulating MAIT cells, as assessed by flow cytometry (**Figure 3B**) and ELISA for IFNγ on purified Vα7.2^+^ T cells (**Supplementary Figure 3F**). In contrast, STAT4 inhibition had no detectable effect.

Next, we focused on discerning the metabolic requirements for IFNγ production under these conditions. MAIT cells have a highly plastic metabolism that adapts to regulate immune cell functions (34, 35), but the effects of IL-12p70 or IL-33 signaling remain unknown. Vα7.2^+^ T cells were treated with the glycolysis inhibitor 2-deoxyglucose (2DG) or the OXPHOS inhibitor oligomycin while stimulation with IL-33 and IL-12p70 (**Figure 3C**), and IFNγ production was assessed through flow cytometry (gating onto MAIT cells, **Figure 3C**) and ELISA (**Supplementary Figure 3G**). Our findings suggest that IFNγ secretion mainly relies on glycolysis, as only 2DG substantially reduced IFNγ production on MAIT cells, being only inhibited on a very much lesser extent (but still significant) by the OXPHOS inhibitor (**Figure 3C**; **Supplementary Figure 3G**).

In conclusion, IL-33 and IL-12p70 induce signaling and metabolic reprograming on MAIT cells, where IFNγ secretion is significantly dependent on p38-MAPK and predominantly glycolysis.

### 3.4 IL-33/IL-12p70 synergistic signaling induces a characteristic protein secretome in human circulating MAIT cells that goes beyond IFNγ

**Figure 4:**
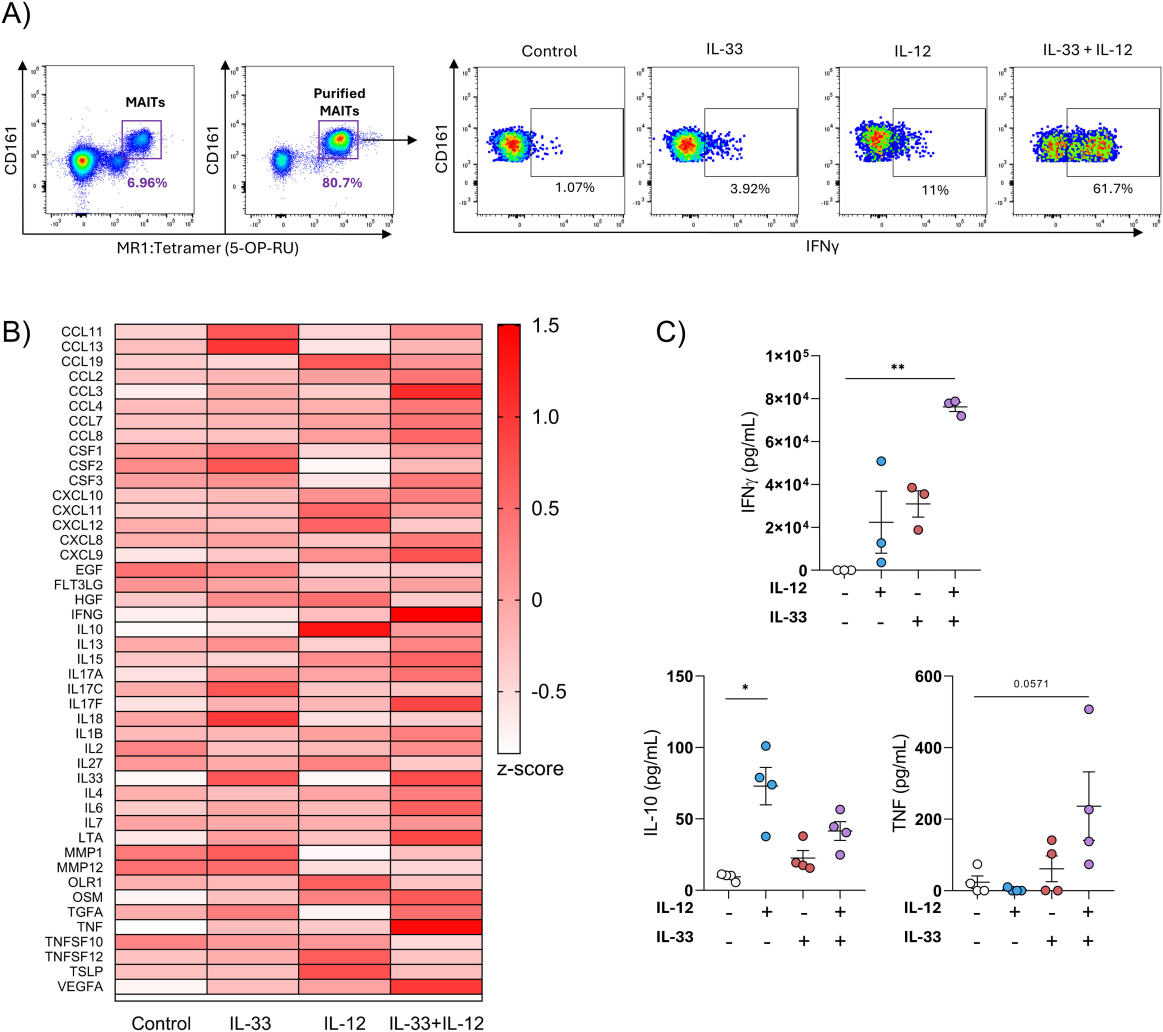
IL-33/IL-12p70 signaling induces a characteristic proinflammatory program in MAIT cells. **(A)** MAIT cells were purified using a 5-OP-RU loaded MR1 tetramer and treated with IL-33 and/or IL-12p70 for 48h (n=4). Representative flow cytometry plots for IFNγ+ MAITs from one of the donors is shown. **(B)** Heatmap of the proteins measured by Olink® proteomics, using z-score protein quantification data from MAITs after each treatment. Red bands indicate higher protein levels, while white bands indicate lower protein levels. **(C)** Bar charts representing cytokine concentration (pg/mL) measured by ELISA assays (IFNγ, IL-10, TNF). ELISA assays were performed using purified Vα7.2+ T cells-derived media. Bars represent mean ± SEM, each dot is an independent donor (n = 4). *P < 0.05, **P < 0.01 Student’s t-test.

Once proven that IL-33/IL-12p70 induce a potent increase in IFNγ production, we aimed to further describe the whole immunomodulatory program induced by both signals. Within this purpose, purified MAIT cells, using a 5-OP-RU-loaded PE-conjugated MR1 tetramer, were cultured in the presence of IL-33, IL-12p70 or both following same conditions as described before (**Figure 4A**, left). As expected, flow plots showed that purified MAIT cells display a robust IFNγ with the presence of IL-33 and IL-12p70 (**Figure 4A**, right). MAIT cell-conditioned media (IL-33+IL-12p70) analysis by Olink^®^ technologies revealed a significant increase in other proinflammatory molecules besides IFNγ including interleukins (e.g, IL-10, IL-17F, TNF), chemokines (e.g., CCL3, CXCL9) and other mediators involved in angiogenesis (VEGF). In addition, IL-33+IL-12p70-derived MAIT cell secretome also showed an increase trend in mediators associated with tumor immunity such as OSM (p = 0.0504) and LTA (p = 0.0835) (**Figure 4B**; **Supplementary Figure 5**). Statistical comparison of concentrations obtained by each of the analyzed molecules (pg/mL; **Supplementary Figure 5**), and their respective calculated z-scores, induced on purified MAIT cells by IL-33, IL-12p70 or both are contained in **Supplementary Table 3** (concentrations, pg/mL) and **Supplementary Table 4** (z-scores). In support to the Olink^®^ data, increased levels of IFNγ, IL-10, and TNF (p = 0.0571) under IL-33+IL-12p70 exposure were confirmed by ELISA performed in supernatants derived from cultures of purified Vα7.2^+^ T cells (**Figure 4C**).

In conclusion, the synergistic signaling induced by IL-33/IL-12p70 in circulating human MAITs entails a deep reprogramming that, besides IFNγ secretion, shapes MAIT cell inflammatory functions.

### 3.5 Media derived from IL-33/IL-12p70 activated Vα7.2^+^ T cells (enriched on MAIT cells) can induce a pro-inflammatory phenotype on CD14^+^ monocytes

**Figure 5:**
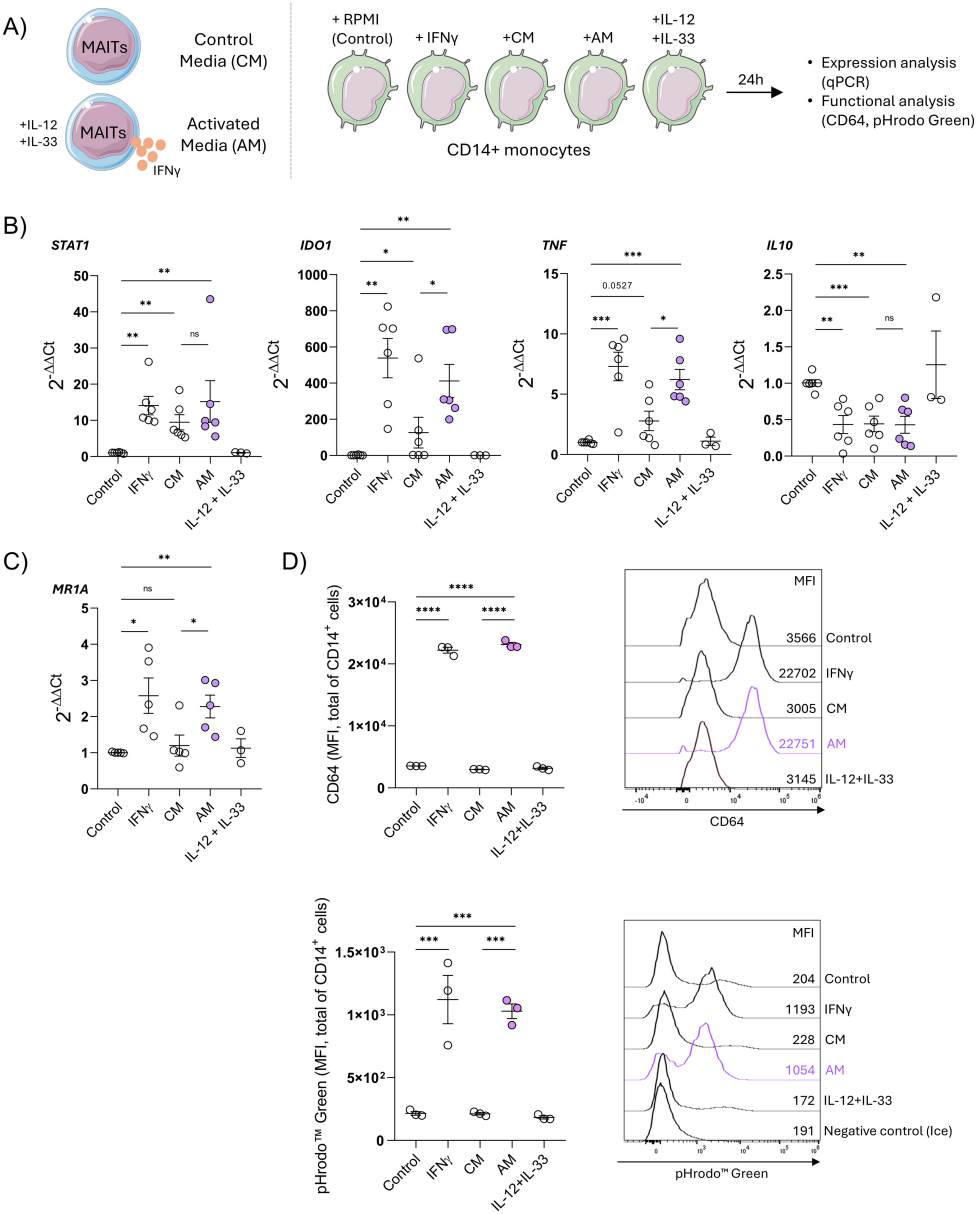
IL-33/IL-12p70-induced MAIT cell-derived media resembles the immunomodulatory features caused by IFNg on CD14+ monocytes. **(A)** Experimental workflow: Vα7.2+ cells were treated with IL-33/IL-12p70 (AM) or in control conditions (CM) for 48h to obtain media derived from activated (AM) and non-activated MAITs (CM), which was later used to culture CD14+ cells for 24h. In all the experiments, control conditions with monocytes cultured in medium alone (RPMI), recombinant IFNγ, or recombinant IL-12p70 + IL-33 were also included in the analysis. Gene expression on CD14+ cells was later analyzed through RT-qPCR and the expression of phagocytosis-related markers was evaluated by flow cytometry. **(B, C)** Bar charts representing the relative mRNA expression (2-ΔΔCt) obtained through RT-qPCR for STAT1, IDO1, TNF, IL-10 and MR1A. 18S was used as a housekeeping gene. **(D)** Bar charts and histograms representing the Median Fluorescence (MFI) for the phagocytosis markers CD64 and pHrodo™ Green. Bars represent mean ± SEM, each dot is biological replicate representing Vα7.2+ T cell-derived media from an independent donor (n = 3-6). ns, not significant; *P < 0.05, **P < 0.01 Student’s t-test **(A–C)** and ANOVA with multiple comparisons test **(D)**. Images contained in this figure were provided by Servier Medical Art (https://smart.servier.com), licensed under CC BY 4.0 (https://creativecommons.org/licenses/by/4.0/).

To evaluate the impact of IL-33/IL-12p70-activated MAIT cells on neighboring innate immune cells, CD14^+^ monocytes were cultured with conditioned media from either non-activated or IL-12p70/IL-33-activated Vα7.2^+^ T cells (enriched on MAIT cells), or with recombinant IFNγ (**Figure 5A**). First, we focused on the possible functions of Vα7.2^+^ T cell-derived IFNγ, since our results show that it is among the most abundant cytokines secreted by MAIT cells in response to IL-12p70/IL-33 (**Supplementary Table 3**). IFNγ is known to polarize monocytes and macrophages towards a proinflammatory state (36–39). CD14^+^ monocytes were cultured with media derived from non-activated and activated (IL-33/IL-12p70) Vα7.2^+^ T cells or recombinant IFNγ. Changes in genes related to IFNγ immunomodulation were evaluated by RT-qPCR (**Figure 5A**). Mechanistically, IFNγ signaling has been extensively described to be mediated by the cytosolic factor STAT1 (40) and to enhance IDO1 production (41). Indeed, our results show that media derived from IL-33/IL-12p70 activated Vα7.2^+^ T cells (AM) generally induced an increase in mRNA expression of *STAT1, IDO1, TNF* on CD14^+^ monocytes, while decreasing the expression of *IL10*, which correlate to the results obtained when treating cells directly with recombinant IFNγ (**Figure 5B**). Importantly, only for *IDO1* and *TNF*, the expression was significantly elevated when comparing AM vs media derived from non-stimulated Vα7.2^+^ T cells (CM). Notably, these effects were not recapitulated by direct cytokine stimulation alone (IL-12+IL-33, cell-free control media), supporting that only Vα7.2^+^ T cell-derived factors contributed to the observed monocyte responses.

IFNγ has also been described to upregulate antigen presenting molecules, including MR1, in other cell types (42). To test whether these conditions upregulate antigen presenting molecules related with MAIT cell biology in CD14^+^ monocytes, the expression of *MR1A* was also evaluated, finding a significant increase when monocytes were treated with media from IL-33/IL-12p70 activated Vα7.2^+^ T cells, similarly to those treated with recombinant IFNγ (**Figure 5C**). Again, AM *vs* CM comparison yielded a significant increase in *MR1A* expression.

Of note, since stimulation with IL-12p70 alone was also able to slightly induce IFNγ production on MAIT cells (**Figure 1C**), we treated CD14^+^ monocytes with media derived from IL-12p70 stimulated Vα7.2+ T cells. Results in **Supplementary Figure 6** show that for all cases except *IL10*, alterations in gene expression after treatment with media from IL-12p70 stimulated Vα7.2+ T cells were comparable to those obtained after treatment with media from IL-33/IL-12p70 stimulated T cells.

We further characterize if activated MAIT cells can modulate monocytes’ phagocytic function. Phagocytosis was measured via CD64 surface expression, a receptor upregulated upon monocyte activation and associated with enhanced phagocytic capacity (43, 44) and the uptake of pHrodo™ Green BioParticles™ (**Figure 5D**). Media from activated Vα7.2^+^ T cells significantly increased both CD64 expression (**Figure 5D**, top) and particle uptake (**Figure 5D**, bottom) with up to six-fold enhancement relative to controls, similar results to those where only recombinant IFNγ was added to the culture. Again, direct cytokine stimulation alone (IL-12 + IL-33, cell-free media control) did not affect monocyte’s phagocytic function. These findings confirm a strong increment of phagocytic capacities caused by IL-33/IL-12p70-activated Vα7.2^+^ T (enriched on MAIT) cells.

In conclusion, our results suggest that IL-33/IL-12p70-activated MAITs can exert immunomodulatory functions on CD14^+^ monocytes, inducing a pro-inflammatory phenotype, upregulating antigen presentation, and enhancing phagocytic capacity that can be potentially mediated by IFNγ, highlighting the potential of MAIT cells to coordinate early immune responses.

## 4 Discussion

MAIT cells constitute a subset of unconventional T lymphocytes whose functions have been predominantly explored in inflammatory contexts during microbial infections, owing to their capacity to recognize both TCR-dependent ligands, such as 5-OP-RU and TCR-independent stimuli, including the cytokines IL-12, IL-18 and type I IFN (24, 45, 46). Despite this well-established role, the involvement of MAIT cells in immune responses initiated by other early immune mediators such as alarmins remains poorly characterized. In this study, we investigated the functional response of circulating MAIT cells to the epithelial-derived alarmins IL-25, IL-33, and TSLP.

Our data demonstrates that, alone, these cytokines (IL-25, IL-33, TSLP) are insufficient to alter MAIT cell frequency, viability, proliferative capacity, activation status, or effector function. We have identified a potent synergistic interaction between IL-33 and the TCR-independent stimuli IL-12p70, that robustly activates MAIT cells and drives their polarization toward a MAIT1 effector phenotype (elevated *TBX21* expression, which encodes for T-bet transcription factor), characterized by markedly enhanced IFNγ production and Granzyme B secretion. Synergistic signaling between IL-33 and IL-12p70 has previously been described in other immune populations with cytotoxic capabilities such as total CD8^+^ T cells and human NK cells, where it promotes effector differentiation and cytokine secretion (33, 47). The biological importance of IL-33 and IL-12 has been previously explored in other pathological scenarios such as COVID-19 infection or breast cancer, where the IL-33/IL-12 ratio played a role in disease severity (48, 49). Furthermore, a recent study by Arrizabalaga et al. (2026) has demonstrated that synergistic effect of IL-33 and IL-12 may have a therapeutic effect on peritoneal carcinomatosis through the increase of IFNγ production (50). Our work here supports and mechanistically expands a recent observation published by Azzout et al. (2021) in MAIT cells, although the underlying mechanisms and the functional scope of this response remained undefined (51). In addition, co-stimulatory effects have also been described for other MAIT cell-related stimuli, such as IL-21, that have been proven to enhance the cytotoxic potential of MAIT cells when combined with TCR -dependent (5-OP-RU) and independent (IL-12/IL-18) signals (52).

Importantly, we show here that both IL-33 and IL-12p70 exert direct effects on MAIT cells, as comparable activation profiles and cytokine induction were observed in total PBMC cultures and in purified MAIT cell populations. In line with this, we observed modulation of both *ST2* (encoding for the IL-33 receptor) and *IL-12RB* gene expression following stimulation with IL-33 and/or IL-12p70, further supporting the notion that MAIT cells are direct targets of these cytokines and consistent with those previously reported by Azzout et al. (2021) (51).

Secretion of IFNγ by circulating MAIT cells has been described in multiple disease onsets such as inflammatory bowel disease (IBD) (53), acute sepsis (54), hepatocellular carcinoma (54, 55). Concretely, IFNγ^+^ MAITs has been described as a potential biomarker of clinical response to allergen immunotherapy in patients with allergic rhinitis, where increased IFNγ expression correlates with improved immunological tolerance (56). More recently, a study by A. M. Cannata et al. has shown that MAIT cell-derived IFNγ could airway hyperresponsiveness by suppressing IgE produced by B cells (57). Along this line, IFNγ secretion from circulating MAIT cells has been found to be specifically inhibited by estrogens in asthmatic patients (and not in healthy individuals) (58). Altogether, these findings underscore the importance of exploring the mechanisms behind MAIT cell-derived IFNγ production and its role as potential regulator of different immune outcomes. Our results demonstrate that IFNγ production in MAIT cells is critically dependent on activation of the p38 MAPK pathway. This pathway has been previously implicated in IL-33-mediated response in other immune populations (59, 60) as well as the regulation of IFNγ production by CD4^+^ T cells and NK cells (47, 61), suggesting that MAIT cells engage conserved T1-like signaling programs in inflammatory environments. In contrast, we were unable to show dependence on STAT4 signaling, contrary to what was previously published in IL-12-induced T cell activation and IFNγ production (62, 63). It is worthy to mention that in a recent publication was also pointed out the difficulties examining STAT4 signaling blockade on MAIT cells using the only commercially available inhibitor Lisofylline, as the effective concentration exceeded the tolerable DMSO levels in culture (64). Therefore, further investigation will be required to unravel how IL-12 contributes to the signaling pathways and final activation of MAIT cells in synergy with IL-33.

In addition, our data reveal a strict dependence of IL-33/IL-12p70-induced IFNγ production from glycolysis. This finding is supported with previous reports indicating that MAIT cell IFNγ production is predominantly glycolysis-dependent through the activation of the mTORC1 signaling pathway (34, 65), highlighting also the metabolic-dependent nature of MAIT cell functions. Notably, we observed a subtle but still significant OXPHOS-dependent IFNγ production by MAIT cells, a fact that has been also reported in other innate-like lymphocyte populations, such as NKT and NK cells (66, 67), suggesting that distinct inflammatory cues can impose alternative bioenergetic demands on effector function. It is therefore conceivable that MAIT cells dynamically could adapt their metabolic programs in response to tissue-specific microenvironments or combinatorial inflammatory signals, a possibility that warrants further investigation.

We next sought to determine the broader extent of MAIT cell functional reprogramming induced by IL33/IL12p70 combination. Beyond their well-established role in antimicrobial host defense, MAIT cells have been implicated in a wide range of immune contexts, including tissue repair, autoimmunity, and cancer (7). A proteomic analysis of the MAIT cell secretome revealed a characteristic protein profile specifically associated with IL-33/IL-12p70 signaling, indicating that this cytokine milieu induces a broad and coordinated functional reprogramming of MAIT cells.

In addition to IFNγ, we observed increased VEGFA and CCL3 expression, suggesting that MAIT cells may contribute to angiogenesis and vascular remodeling (68). Together with previous evidence that activated MAIT cells express trafficking receptors linked to endothelial interactions and extravasation, our findings suggest that IL-33/IL-12p70-driven inflammation may also impact endothelial function and vascular dynamics (69). Future studies will be required to dissect whether MAIT cell-derived factors induced by IL-33/IL-12p70 directly modulate endothelial activation, permeability, or vascular remodeling in inflamed tissues. In addition, IL-33/IL-12p70 stimulation on MAIT cells also resulted in increased secretion of the proinflammatory cytokines OSM and TNF, both of which play critical roles in immune-mediated pathology across a broad spectrum of diseases, such as cancer (70, 71). Moreover, TNF, apart from reinforcing this T1-like response on MAIT cells, is a well-established activator of endothelial cells, and its secretion was enhanced in circulating MAIT cells in the presence of IL-33 and IL-12p70, further reinforcing the potential modulatory role of IL-33/IL-12–activated MAIT cells in endothelial biology, as discussed above (69). In conclusion, the induction of all these mediators further supports the notion that IL-33 and IL-12p70 license MAIT cells to adopt a multifunctional inflammatory effector profile that extends beyond canonical antimicrobial responses. Collectively, these observations highlight the potential relevance of IL-33/IL-12p70-driven MAIT cell activation in non-infectious inflammatory settings and warrant further investigation into their contribution to tissue-specific inflammation, chronic immune disorders, and tumor-associated immune responses.

Given this extensive reprogramming of the MAIT cell secretome in response to IL-33 and IL-12p70, we next sought to determine whether these changes translated into functional consequences for neighboring innate immune populations. To this end, we assessed the impact of conditioned media derived from IL-33/IL-12p70-activated Vα7.2^+^ T cells (enriched on MAIT cells) on circulating CD14^+^ monocytes. Monocytes are very abundant in the human blood and are central mediators of innate immunity and play a pivotal role throughout the inflammatory response, from its initiation to its resolution, displaying a high degree of sensitivity to environmental proinflammatory cues (72). Accordingly, monocytes undergo extensive transcriptional and functional reprogramming in response to inflammatory cytokines present within their microenvironment.

IFNγ is a well-established proinflammatory cytokine known to drive monocyte polarization toward a classically activated, proinflammatory state through activation of the JAK/STAT1 signaling axis (38, 73). Consistent with this paradigm, our data suggest that conditioned media from IL-33/IL-12p70–activated Vα7.2^+^ T cells tend to induce a proinflammatory program in monocytes, characterized by an increase on *STAT1* and *IDO1* expressions, gene upregulation of proinflammatory mediators such as *TNF*, and concomitant downregulation of anti-inflammatory genes including *IL10*. Nevertheless, the directional consistency of these effects across donors suggests that Vα7.2^+^ T cells (and potentially MAIT cells) activated by IL-33 and IL-12p70 may exert biologically relevant influences on surrounding innate immune cells through their secretory output. Notably, IFNγ has previously been shown to enhance MR1 transcription in immune and non-immune cells such as the airway epithelial cells, thereby increasing the capacity for MAIT cell antigen presentation and establishing a feed-forward loop between IFNγ-producing cells and MAIT cell activation (42). In line with this, we observed a significant increase in *MR1A* gene expression in monocytes exposed to media from IL-33/IL-12p70-activated Vα7.2^+^ T cells. Our data suggests that MAIT cell-derived IFNγ may amplify monocyte crosstalk by enhancing MR1-dependent antigen presentation, potentially reinforcing MAIT cell activation in inflammatory settings. While IFNγ recapitulates key aspects of the observed transcriptional response, we cannot exclude contributions from other cytokines present in MAIT cell–conditioned media. Future studies using neutralization approaches will be necessary to dissect the relative contribution of individual inflammatory mediators.

Finally, we observed that media from IL-33/IL-12p70-activated Vα7.2^+^ T cells induced CD64 expression and phagocytosis rate. Based on the literature, not only IFNγ but also other immune mediators including CCL3 or TNF have been described to enhance phagocytic capacities (44, 74, 75). Similarly, IFNγ has been proven to increase the expression of high affinity receptor for IgG (FcγRI/CD64) in human monocytes (76), which results in enhanced phagocytic functions (77). These findings support what was observed in our study and suggest a relevant role for MAIT cell–derived mediators in the control of phagocytosis. However, as the Vα7.2^+^ T cell population also contains non-MAIT T cells, the potential contribution of other cells (besides MAIT cells) to the observed effects cannot be excluded and will require further investigation.

Together, these data support the rationale of the synergistic IL-33/IL-12p70 promotion of MAIT cells function as potent immunomodulatory effectors capable of shaping the inflammatory landscape beyond their direct activation. Through IFNγ (and other proinflammatory cytokines) driven paracrine signaling, activated MAIT cells can promote proinflammatory programming in monocytes and potentially influence broader immune networks. While the present study focuses on the IFNγ secretion by MAIT cells using monocytes as a proof-of-concept target population, further investigation will be required to determine the contribution of this cytokine and other MAIT cell–derived signals in monocyte cell biology, and their potential to modulate other immune subsets, including macrophages, dendritic cells, and conventional T cells, in both tissue-specific and disease-relevant contexts.

## Data Availability

The datasets generated and analyzed during the current study are provided in the **Supplementary Materials** (**Supplementary Figure 5**; **Supplementary Table 3**), including individual concentration values (pg/mL) for all analyzed proteins measured using the Olink Target 48 Cytokine panel. The affinity proteomics data have been deposited to the PRIDE (PRoteomics IDEntifications Database) (78) repository with the dataset identifier PAD000044.
