# Supplementary material for: Synergistic TCR-independent action of IL-33 and IL-12 drive a potent IFNγ secretory program in human circulating MAIT cells with immunomodulatory properties
Source: Front Immunol. 2026 Jun 26;17:1852383. doi: 10.3389/fimmu.2026.1852383 (PMC13349841; doi:10.3389/fimmu.2026.1852383)
Supplement: Supplementary file 1 [file DataSheet1.docx]

Supplementary Material

**
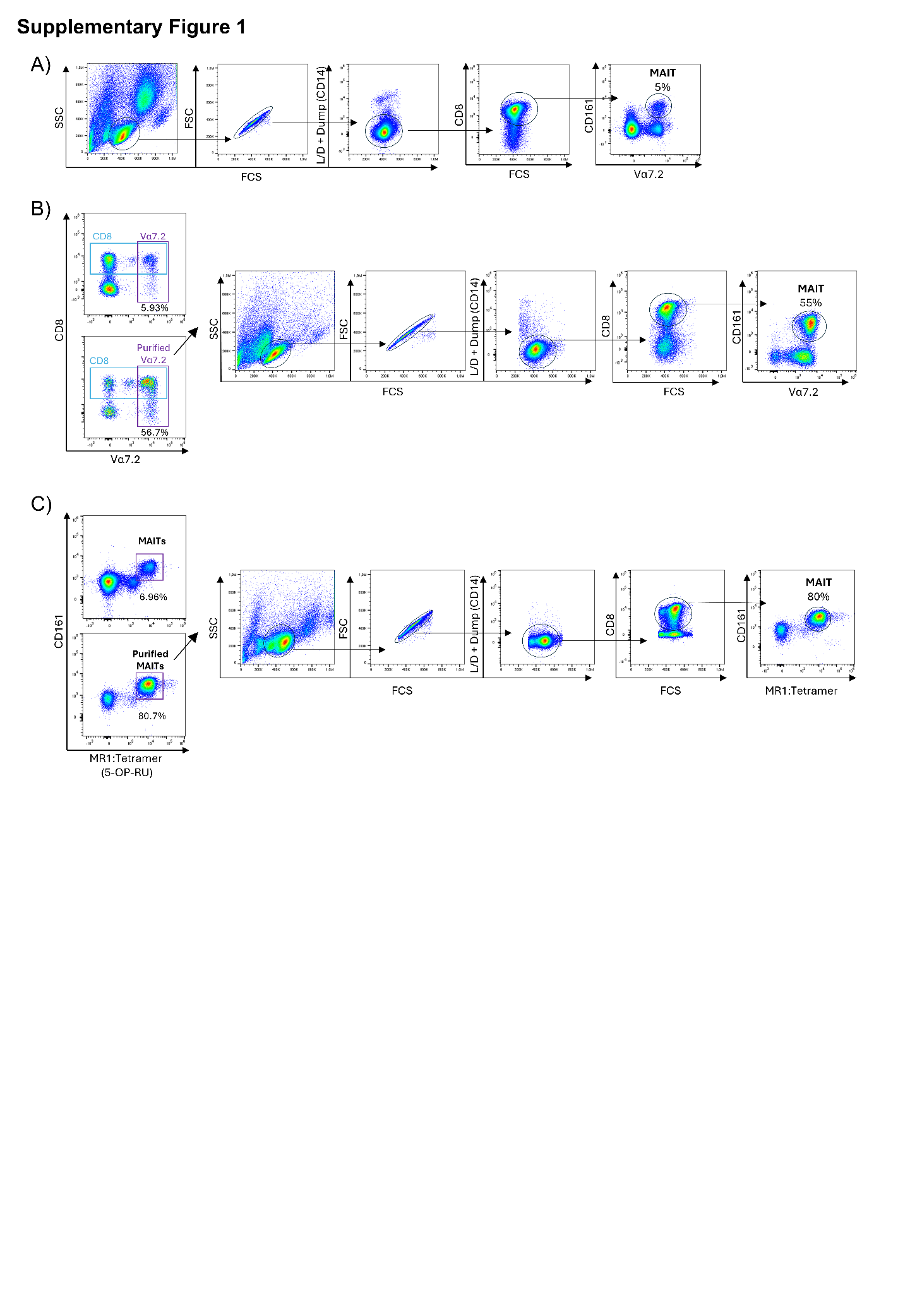
**

**Supplementary Figure 1.** Flow cytometry plots representing the fractions cultured and MAIT cells’ gating along the study. (A) Among PBMCs, MAIT cells were gated as Zombie^−^CD14^−^CD8α^+^CD161^+^Vα7.2^+^ cells, as shown in the cell gating example flow plots. (B) Purified Vα7.2^+^ cells and (C) Purified MAIT cells. MAIT cells were gated as Zombie^−^CD14^−^CD8α^+^CD161^+^Vα7.2^+^ cells or Zombie^−^CD14^−^CD8α^+^CD161^+^MR1: Tetramer^+^, respectively.


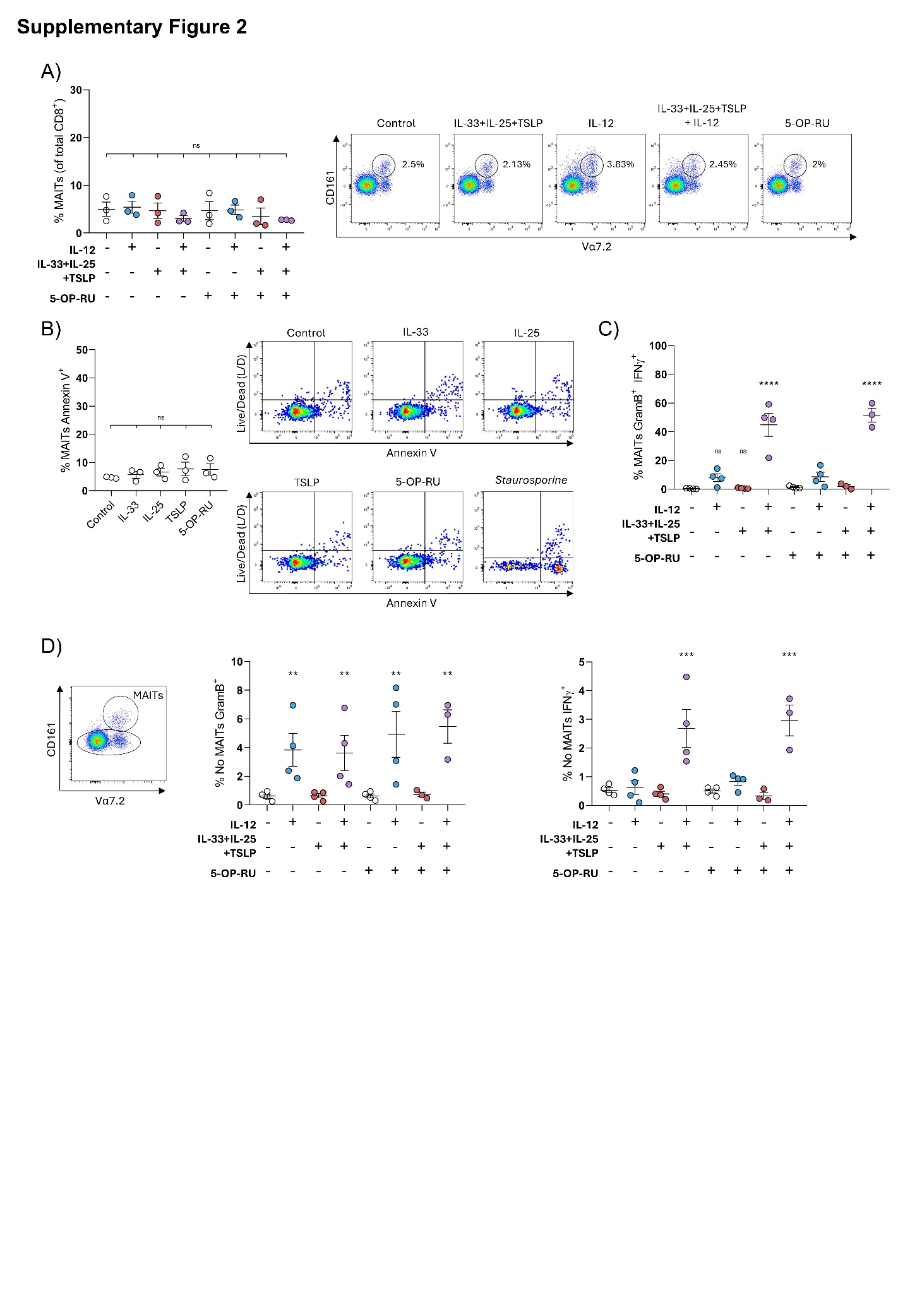


**Supplementary Figure 2.** Whole PBMCs were cultured for 48h in the presence of IL-25, IL-33 and TSLP (individually or together), 5-OP-RU and IL-12p70. (A) Bar charts and representative flow cytometry plots represent the percentage of MAITs after treatment. (B) Viability assay: Bar charts and representative flow plots for the percentage of MAITs Annexin V^+^. For this assay, a positive control for apoptosis was included (Staurosporine, *Streptomyces sp.* 1µM, 6 hours). (C) Bar chart for the percentage of double positive GramB^+^ IFNγ^+^ MAIT cells. (D) Evaluation of production of GramB and IFNγ in CD8^+^ No-MAIT cells. Left part of each section shows a representative flow cytometry plot of the population analyzed and right part includes bar charts for the percentage of GramB^+^ and IFNγ^+^ cells. Bars represent Mean ± SEM, each dot is an independent donor (n = 3-4). All comparisons are made with respect to the unstimulated condition. ns, not significant; **P < 0.01, ***P<0.001.****P < 0.0001 ANOVA with multiple comparisons test.


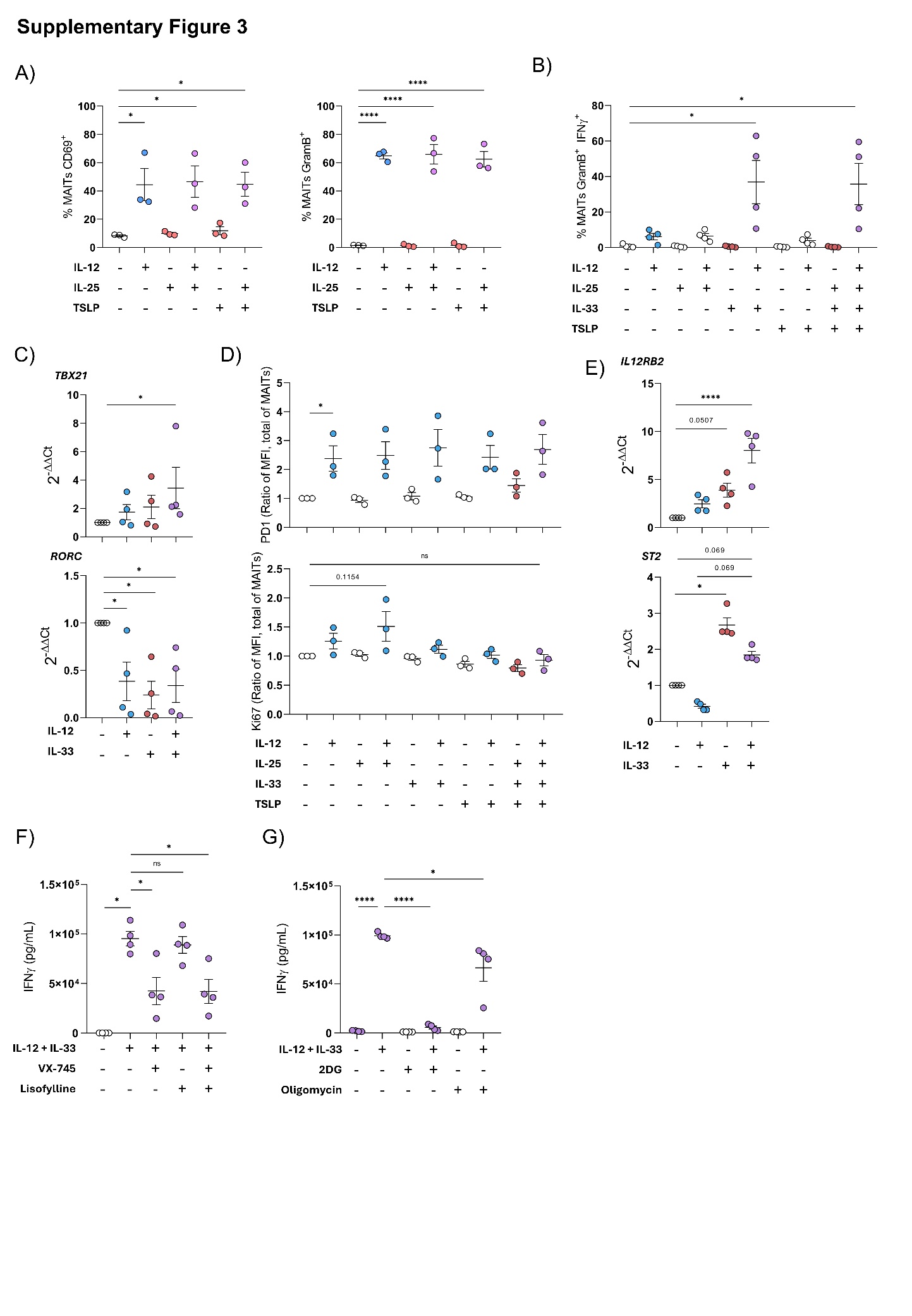


**Supplementary Figure 3.** Whole PBMCs were cultured for 48h in the presence of each alarmin (IL-25, IL-33, TSLP), all three together and in combination with IL-12p70. (A) Bar charts for the percentage of CD69^+^ and GramB^+^ MAIT cells. (B) Bar chart for the percentage of double positive GramB^+^ IFNγ^+^ MAIT cells. (C) Bar charts representing the relative expression (2^-∆∆Ct^) obtained through RT-qPCR for *TBX21* (encoding for T-bet) and *RORC* (encoding for RORγt) on purified Vα7.2^+^ cells. (D) Bar charts with the Mean Fluorescence Intensity (MFI) for the markers PD1 and Ki67 of the total population of MAIT cells after the indicated conditions. (E) Bar charts representing the relative expression (2-∆∆Ct) obtained through RT-qPCR for *ST2* (encoding for IL-33R) and *IL12RB2* (encoding for IL-12R) on purified Vα7.2^+^ cells. (F) Purified Vα7.2+ cells were treated for 48h with IL-33/IL-12p70 in combination with VX-745 (P38 inhibitor) and/or Lisofylline (STAT4 inhibitor). (G) Purified Vα7.2+ cells were treated for 48h with IL-33/IL-12p70 in combination with 2-DG (Glycolysis inhibitor) or Oligomycin (OXPHOS inhibitor). For F-G, bar chart represents the concentration (pg/mL) of IFNγ obtained by ELISA assays. Bars represent Mean ± SEM, each dot is an independent donor (n = 3-4). ns, not significant; *P < 0.05, ****P < 0.0001 ANOVA with multiple comparisons test (A, B, D), and Student's t-test (C, E, F, G).


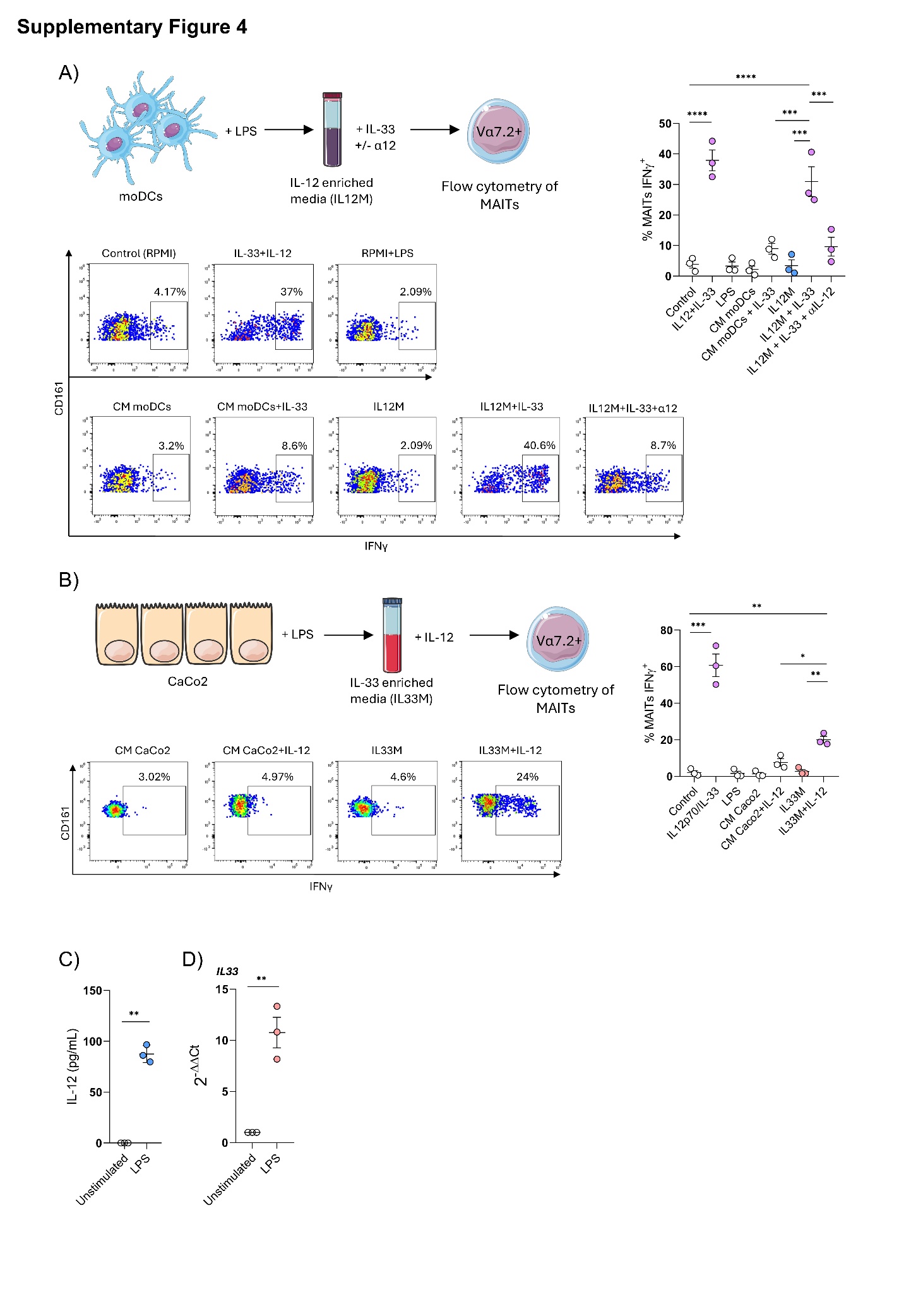


**Supplementary Figure 4.** Biological validation of IFNγ secretion by MAIT cells with IL-33- and IL-12- producing cells. For each dataset, the experimental workflow is shown at the top. (A) moDCs were treated with LPS (250 ng/mL) for 24h to obtain IL-12p70-enriched culture media (IL12M) which was used with or without the combination of IL-33, and/or anti-IL-12p70 blocking antibody (αIL-12p70) to culture Vα7.2^+^ T cells for 48h. (B) CaCo2 cells were treated with LPS (2μg/mL) for 24h to obtain IL-33 enriched culture media (IL33M), which was used in combination with IL-12p70 to culture Vα7.2^+^ T cells for 48h. Flow cytometry analysis was done on MAIT cell compartment. Conditions: CM = Media obtained from unstimulated cells (either moDCs or Caco2 cells), IL12M = Media from activated moDCs rich in IL-12, IL33M = Media from activated Caco2 rich in IL-33. The figure includes bar charts and representative flow cytometry plots for IFNγ^+^ MAIT cells. C) IL-12p70 levels (pg/mL) in IL12M derived from LPS-stimulated moDCs were quantified by ELISA (one representative experiment). D) Relative expression of *IL33* gene (2^-∆∆Ct^) was analyzed by RT-qPCR on Caco-2 cell lysates after LPS stimulation (one representative experiment). Bars represent mean ± SEM, each dot is an independent donor (n = 3). *P < 0.05, **P < 0.01, ***P < 0.001, ****P < 0.0001 ANOVA with multiple comparisons test (A, B) and Student’s T test (C, D). Images contained in this figure were provided by Servier Medical Art (<https://smart.servier.com>), licensed under CC BY 4.0 (https://creativecommons.org/licenses/by/4.0/).


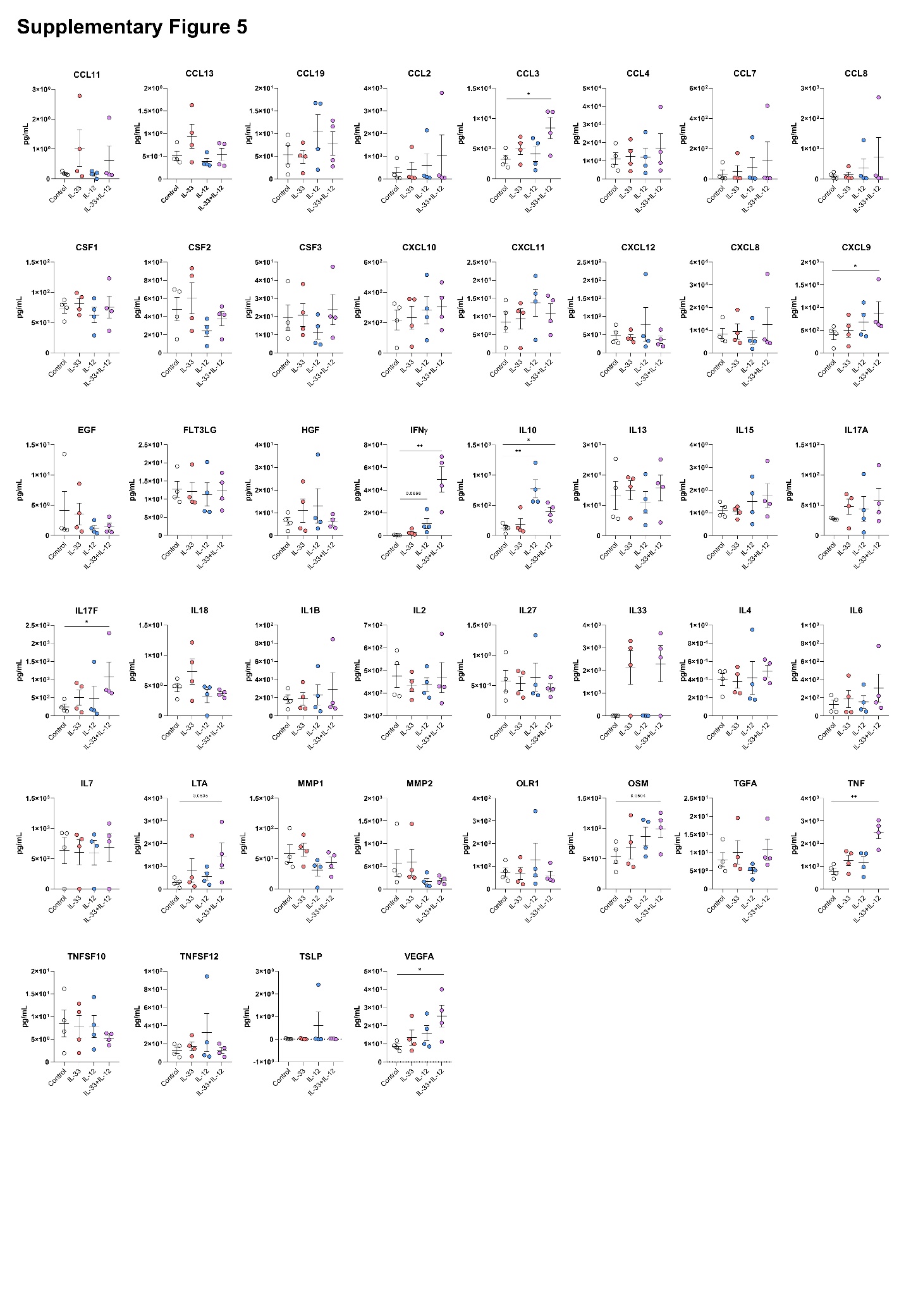


**Supplementary Figure 5.**  Purified MAIT cells were cultured with IL-33 and/or IL-12p70 for 48h. Supernatants were analyzed by Olink® technologies. The figure shows bar charts with statistical comparisons of concentrations obtained by each of the analyzed molecules. Bars represent mean ± SEM, each dot is an independent donor (n = 4). *P < 0.05, **P < 0.01 Student's t-test or Mann-Whitney U test.

**
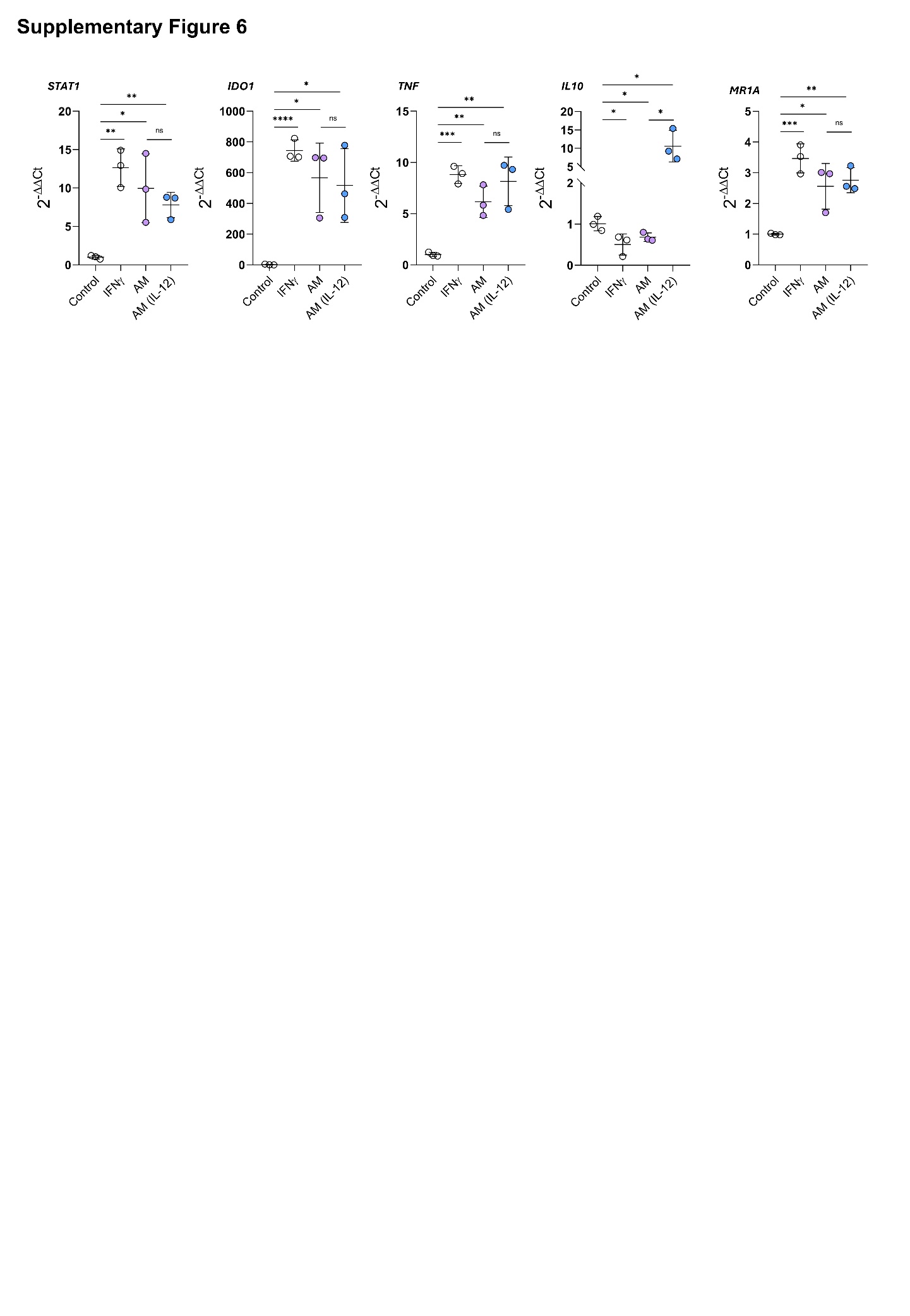
**

**Supplementary Figure 6.** Purified CD14^+^ monocytes were treated for 24h with recombinant IFNγ, media derived from IL-33/IL-12p70 activated MAITs (AM IL-33+IL-12) or IL-12p70 activated MAITs (AM IL-12). After treatment, *STAT1, IDO1, TNF, IL10* and *MR1A* relative (2^-∆∆Ct^) gene expression was analyzed through RT-qPCR. Figure includes bar charts for each gene analyzed. Bars represent mean ± SEM, each dot is an independent donor (n = 3). ns, not significant, *P < 0.05, **P < 0.01, ***P < 0.001, ****P < 0.0001 Student's t-test.

**Supplementary Table 1. List of antibodies and reagents used for Flow Cytometry staining.**

| **Marker** | **Fluorochrome** | **Clone** | **Cat No.** | **Brand** |
| --- | --- | --- | --- | --- |
| Annexin V | FITC | - | 640922 | Biolegend |
| CD8 | PERCP | SK1 | 344707 | Biolegend |
| CD14 | BV421 | 63D3 | 367144 | Biolegend |
| CD19 | Pacific Blue | SJ25C1 | 363035 | Biolegend |
| CD64 | Pecy7 | S18012C | 399507 | Biolegend |
| CD69 | BV510 | FN50 | 310935 | Biolegend |
| CD161 | AF647 | HP-3G10 | 339910 | Biolegend |
| GRAMB | FITC | GB11 | 515403 | Biolegend |
| IFNγ | BV650 | 4S.B3 | 502537 | Biolegend |
| pHrodo™ Green-E. coli BioParticles™ | | - | P35366 | Invitrogen |
| Vα7.2 | PE | 3C10 | 351705 | Biolegend |
| Zombie | Near-IR | - | 423105 | Biolegend |

**Supplementary Table 2. List of oligonucleotides (5’-3’) used for RT-qPCR.**

| **Gen** | **Primer Forward** | **Primer Reverse** |
| --- | --- | --- |
| *18S* | ATCGGGGATTGCAATTATTC | CTCACTAAACCATCCAATCG |
| *IDO1* | CTCCTGGACAATCAGTAAAGAGTACC | ACTTCTCAACTCTTTCTCGAAGCTGG |
| *IL10* | TCTCCGAGATGCCTTCAGCAGA | TCAGACAAGGCTTGGCAACCCA |
| *IL12RB2* | GTTGGAGTGATTGGAGTG | CCTGTGATGTTCTGTGTC |
| *IL33* | GGTGTTGATGGTAAGATGTT | AGAGTGTTCCTTGTTGTTG |
| *MR1A* | AGGGGTTACAGCTCTCTTCTG | TTGATGCCCACGCCTG |
| *RORC* | GAGGAAGTGACTGGCTACCAGA | GCACAATCTGGTCATTCTGGCAG |
| *ST2* | GGATTGAGGCCACTCTGCTC | CCGCCTGCTCTTTCGTATGT |
| *STAT1* | AATTTTCCCTTCTGGCTTTGG | TCTCGCTCCTTGCTGATGAA |
| *TBX21* | CCACCTGTTGTGGTCCAAGT | AATGGGAACATCCGCCGTC |
| *TNF* | AACCTCCTCTCTGCCATCAA | GGAAGACCCCTCCCAGATAG |

**Supplementary Table 3. Changes induced in the secretome of MAIT cells by IL-33 and IL-12p70 (pg/mL).**

|  | **Control** | | | | **IL-33** | | | | **IL-12** | | | | **IL-33+IL-12** | | | | **No cells** | | | |
| --- | --- | --- | --- | --- | --- | --- | --- | --- | --- | --- | --- | --- | --- | --- | --- | --- | --- | --- | --- | --- |
| **pg/mL** | **Donor 1** | **Donor 2** | **Donor 3** | **Donor 4** | **Donor 1** | **Donor 2** | **Donor 3** | **Donor 4** | **Donor 1** | **Donor 2** | **Donor 3** | **Donor 4** | **Donor 1** | **Donor 2** | **Donor 3** | **Donor 4** | **Control** | **IL33** | **IL12** | **IL33-IL12** |
| **CCL11** | 0.17 | 0.13 | 0.27 | 0.18 | 0.26 | 2.78 | 1.00 | 0.09 | 0.20 | 0.26 | 0.00 | 0.16 | 2.05 | 0.13 | 0.14 | 0.19 | 0.00 | 0.00 | 0.00 | 0.00 |
| **CCL13** | 0.37 | 0.43 | 0.44 | 0.81 | 0.37 | 1.63 | 1.02 | 0.75 | 0.33 | 0.30 | 0.32 | 0.59 | 0.77 | 0.29 | 0.32 | 0.79 | 0.05 | 0.05 | 0.03 | 0.04 |
| **CCL19** | 7.59 | 9.73 | 3.01 | 0.98 | 8.00 | 5.15 | 4.98 | 1.26 | 16.63 | 16.72 | 6.57 | 2.02 | 11.58 | 12.88 | 2.76 | 4.15 | 0.02 | 0.05 | 0.01 | 0.07 |
| **CCL2** | 29.13 | 174.29 | 55.47 | 929.31 | 41.24 | 92.74 | 68.13 | 1412.74 | 55.99 | 149.64 | 87.52 | 2140.32 | 47.38 | 153.75 | 63.35 | 3794.19 | 0.26 | 0.32 | 0.43 | 0.35 |
| **CCL3** | 5145.82 | 1905.23 | 2397.77 | 3502.64 | 6962.94 | 2350.52 | 5907.08 | 4833.01 | 6750.44 | 1436.31 | 2511.18 | 5906.43 | 11144.54 | 3829.92 | 7614.41 | 11103.11 | 0.00 | 0.00 | 0.06 | 0.17 |
| **CCL4** | 12679.37 | 4702.33 | 7348.95 | 19716.52 | 15327.20 | 4236.90 | 8466.71 | 21859.06 | 12177.45 | 3276.82 | 7367.81 | 25791.58 | 14927.27 | 4683.20 | 8888.73 | 39727.67 | 0.00 | 0.00 | 0.05 | 0.29 |
| **CCL7** | 3.51 | 13.96 | 5.46 | 110.80 | 6.95 | 7.72 | 4.40 | 171.34 | 5.29 | 9.62 | 2.88 | 275.18 | 4.22 | 8.75 | 4.42 | 483.92 | 0.00 | 0.00 | 0.00 | 0.00 |
| **CCL8** | 13.78 | 137.51 | 21.22 | 230.88 | 30.16 | 57.48 | 33.73 | 419.09 | 30.43 | 102.64 | 21.24 | 1284.43 | 25.50 | 112.86 | 28.62 | 2695.99 | 0.00 | 0.00 | 0.00 | 0.00 |
| **CSF1** | 87.50 | 51.77 | 74.66 | 79.44 | 99.22 | 62.45 | 71.97 | 92.05 | 90.02 | 28.76 | 59.75 | 71.74 | 68.91 | 36.21 | 73.70 | 123.15 | 0.00 | 0.00 | 0.00 | 0.00 |
| **CSF2** | 67.58 | 15.10 | 39.95 | 69.88 | 85.02 | 24.09 | 38.49 | 93.38 | 28.56 | 7.66 | 37.65 | 22.51 | 51.30 | 14.94 | 42.91 | 42.18 | 0.00 | 0.00 | 0.02 | 0.01 |
| **CSF3** | 16.70 | 7.93 | 39.47 | 13.37 | 9.85 | 13.25 | 22.26 | 37.98 | 14.65 | 5.45 | 4.59 | 21.22 | 19.43 | 8.37 | 20.11 | 47.51 | 0.00 | 0.65 | 0.00 | 0.00 |
| **CXCL10** | 300.44 | 325.68 | 214.18 | 32.68 | 356.56 | 179.46 | 354.16 | 40.60 | 516.41 | 293.25 | 236.52 | 83.64 | 467.03 | 364.11 | 238.81 | 150.89 | 0.00 | 0.00 | 0.00 | 0.00 |
| **CXCL11** | 11.06 | 14.51 | 6.79 | 1.36 | 13.70 | 11.49 | 11.06 | 1.25 | 16.32 | 21.19 | 14.15 | 3.56 | 15.75 | 14.46 | 8.80 | 4.88 | 0.00 | 0.00 | 0.00 | 0.00 |
| **CXCL12** | 29.90 | 77.43 | 56.18 | 26.68 | 29.32 | 63.95 | 39.21 | 38.72 | 45.73 | 17.28 | 217.27 | 33.06 | 63.86 | 17.86 | 23.67 | 36.97 | 0.00 | 0.00 | 4.44 | 4.99 |
| **CXCL8** | 7090.79 | 5149.68 | 5671.09 | 15727.01 | 8731.97 | 4366.39 | 5681.17 | 19030.12 | 5337.90 | 1810.93 | 5065.08 | 15385.08 | 4743.33 | 4305.57 | 5926.24 | 34853.43 | 0.04 | 0.05 | 0.15 | 0.27 |
| **CXCL9** | 584.58 | 484.59 | 428.11 | 97.10 | 843.25 | 404.40 | 594.74 | 144.52 | 1112.05 | 403.49 | 837.30 | 364.54 | 1625.97 | 588.45 | 626.37 | 685.50 | 0.00 | 0.00 | 0.00 | 0.00 |
| **EGF** | 1.18 | 0.91 | 1.01 | 13.47 | 1.33 | 8.55 | 3.65 | 0.67 | 1.19 | 0.61 | 0.38 | 2.55 | 3.08 | 0.52 | 0.64 | 1.61 | 0.00 | 0.00 | 0.00 | 0.00 |
| **FLT3LG** | 12.09 | 9.25 | 19.05 | 10.53 | 10.55 | 9.28 | 19.60 | 9.09 | 11.77 | 6.70 | 20.24 | 6.47 | 10.21 | 6.88 | 17.27 | 14.57 | 0.00 | 0.00 | 0.00 | 0.00 |
| **HGF** | 5.63 | 7.05 | 10.24 | 1.99 | 3.19 | 14.90 | 23.88 | 2.10 | 5.99 | 7.72 | 35.67 | 3.09 | 6.93 | 3.97 | 9.52 | 3.35 | 0.03 | 0.00 | 0.05 | 0.02 |
| **IFNG** | 614.07 | 352.44 | 860.08 | 195.10 | 2366.78 | 964.42 | 6166.69 | 1915.54 | 7859.69 | 2867.75 | 23347.80 | 7766.72 | 64188.51 | 43937.49 | 69559.31 | 20600.61 | 0.00 | 0.00 | 0.56 | 0.35 |
| **IL10** | 206.28 | 146.36 | 126.16 | 26.12 | 126.11 | 85.66 | 468.05 | 69.72 | 561.79 | 560.53 | 760.23 | 1206.59 | 236.58 | 339.59 | 466.99 | 545.99 | 0.00 | 0.10 | 0.24 | 0.01 |
| **IL13** | 15.78 | 6.19 | 25.37 | 5.55 | 19.24 | 18.79 | 16.19 | 5.65 | 20.28 | 8.05 | 12.08 | 3.38 | 25.17 | 16.11 | 17.34 | 4.34 | 0.00 | 0.13 | 0.00 | 0.20 |
| **IL15** | 1.26 | 1.49 | 0.83 | 0.89 | 1.17 | 1.27 | 0.71 | 1.02 | 1.86 | 1.03 | 0.50 | 2.60 | 1.40 | 1.52 | 0.84 | 3.29 | 0.00 | 0.00 | 0.00 | 0.00 |
| **IL17A** | 26.00 | 27.88 | 29.34 | 26.15 | 11.50 | 61.59 | 48.54 | 68.20 | 27.66 | 41.75 | 4.92 | 101.58 | 23.80 | 52.78 | 38.39 | 116.09 | 0.14 | 0.13 | 0.11 | 0.21 |
| **IL17C** | 0.01 | 0.00 | 0.00 | 0.00 | 0.00 | 0.05 | 0.00 | 0.00 | 0.00 | 0.00 | 0.00 | 0.00 | 0.00 | 0.00 | 0.00 | 0.00 | 0.00 | 0.00 | 0.00 | 0.00 |
| **IL17F** | 125.97 | 154.16 | 231.44 | 466.65 | 94.24 | 204.84 | 806.38 | 904.84 | 155.23 | 177.98 | 59.42 | 1492.98 | 678.68 | 625.28 | 711.75 | 2283.01 | 0.61 | 0.59 | 0.78 | 0.56 |
| **IL18** | 6.14 | 4.95 | 4.83 | 2.73 | 5.72 | 12.13 | 8.87 | 2.47 | 4.81 | 4.69 | 0.00 | 3.58 | 4.34 | 3.46 | 2.96 | 3.89 | 0.00 | 0.00 | 0.00 | 0.00 |
| **IL1B** | 16.28 | 7.18 | 19.18 | 30.35 | 8.69 | 8.19 | 20.96 | 37.33 | 9.47 | 5.10 | 22.16 | 54.91 | 9.83 | 8.17 | 14.41 | 84.56 | 3.43 | 0.30 | 2.82 | 0.33 |
| **IL2** | 387.30 | 532.98 | 588.23 | 394.18 | 451.24 | 370.66 | 415.06 | 494.45 | 401.53 | 380.58 | 518.64 | 449.00 | 430.64 | 661.76 | 427.41 | 356.26 | 695.15 | 658.01 | 659.94 | 787.03 |
| **IL27** | 0.40 | 1.05 | 0.61 | 0.25 | 0.71 | 0.74 | 0.37 | 0.30 | 0.51 | 0.37 | 1.33 | 0.34 | 0.64 | 0.44 | 0.31 | 0.45 | 0.00 | 0.00 | 0.00 | 0.00 |
| **IL33** | 5.41 | 4.74 | 8.73 | 0.25 | 3303.73 | 2244.51 | 2989.83 | 0.01 | 6.78 | 15.80 | 4.14 | 0.04 | 3641.86 | 2577.25 | 2964.47 | 0.20 | 0.00 | 2381.36 | 0.08 | 2828.39 |
| **IL4** | 0.49 | 0.41 | 0.49 | 0.21 | 0.26 | 0.46 | 0.54 | 0.25 | 0.35 | 0.19 | 0.95 | 0.18 | 0.41 | 0.62 | 0.58 | 0.36 | 0.00 | 0.00 | 0.00 | 0.00 |
| **IL6** | 46.53 | 49.85 | 227.60 | 179.93 | 43.73 | 41.73 | 212.97 | 445.51 | 70.99 | 48.42 | 148.25 | 346.71 | 147.59 | 88.80 | 220.78 | 770.22 | 0.03 | 0.01 | 0.00 | 0.01 |
| **IL7** | 687.28 | 919.61 | 925.54 | 0.21 | 894.33 | 704.38 | 825.65 | 0.07 | 782.66 | 712.84 | 899.77 | 0.00 | 784.33 | 1084.66 | 876.43 | 0.00 | 1550.80 | 1463.30 | 1738.63 | 1494.29 |
| **LTA** | 327.17 | 245.37 | 505.61 | 52.84 | 503.72 | 297.30 | 2352.15 | 113.59 | 687.87 | 356.58 | 994.77 | 188.37 | 1547.55 | 1051.86 | 2957.48 | 292.73 | 0.00 | 0.02 | 0.04 | 0.02 |
| **MMP1** | 100.58 | 36.26 | 43.21 | 52.90 | 89.69 | 37.09 | 62.13 | 69.97 | 47.62 | 1.94 | 38.49 | 36.61 | 55.48 | 19.88 | 37.84 | 60.55 | 0.00 | 0.00 | 0.00 | 0.00 |
| **MMP12** | 1439.26 | 408.78 | 301.95 | 153.04 | 1434.11 | 414.13 | 248.43 | 272.71 | 365.23 | 76.40 | 163.48 | 64.76 | 295.36 | 139.71 | 228.61 | 103.36 | 0.00 | 0.00 | 0.00 | 0.00 |
| **OLR1** | 51.90 | 78.77 | 127.01 | 36.15 | 32.71 | 80.77 | 140.23 | 21.13 | 60.74 | 88.76 | 343.59 | 23.77 | 36.42 | 46.36 | 115.84 | 41.26 | 0.00 | 0.00 | 0.00 | 0.00 |
| **OSM** | 65.45 | 44.55 | 78.26 | 27.85 | 77.17 | 36.19 | 121.95 | 41.50 | 114.47 | 53.60 | 111.05 | 68.31 | 101.35 | 57.32 | 113.48 | 126.00 | 0.00 | 0.00 | 0.00 | 0.00 |
| **TGFA** | 7.50 | 6.10 | 13.69 | 4.92 | 8.75 | 6.49 | 19.58 | 5.49 | 6.93 | 5.09 | 2.58 | 5.49 | 8.66 | 6.68 | 19.41 | 8.43 | 2.28 | 1.93 | 2.49 | 2.42 |
| **TNF** | 1102.68 | 449.35 | 689.13 | 918.50 | 1571.68 | 661.63 | 1667.75 | 1131.29 | 1581.48 | 533.26 | 973.32 | 1561.44 | 3031.96 | 1723.91 | 2485.15 | 2782.58 | 0.48 | 0.32 | 0.21 | 0.44 |
| **TNFSF10** | 9.18 | 16.10 | 6.72 | 1.92 | 4.94 | 12.86 | 11.00 | 1.98 | 8.17 | 6.00 | 14.33 | 2.74 | 6.30 | 4.91 | 6.18 | 3.71 | 0.00 | 0.00 | 0.00 | 0.00 |
| **TNFSF12** | 17.78 | 9.15 | 19.77 | 5.32 | 14.57 | 17.86 | 29.40 | 6.15 | 21.54 | 7.56 | 94.37 | 5.92 | 15.41 | 5.62 | 20.12 | 9.90 | 0.00 | 0.00 | 0.00 | 0.00 |
| **TSLP** | 0.00 | 0.00 | 0.05 | 0.00 | 0.05 | 0.01 | 0.00 | 0.00 | 0.00 | 0.02 | 2.41 | 0.00 | 0.03 | 0.03 | 0.02 | 0.00 | 0.03 | 0.00 | 0.01 | 0.00 |
| **VEGFA** | 8.92 | 7.91 | 11.74 | 5.98 | 9.85 | 12.42 | 25.48 | 6.24 | 18.23 | 9.95 | 26.68 | 8.56 | 28.43 | 21.50 | 39.98 | 11.12 | 0.09 | 0.06 | 0.05 | 0.06 |

**Supplementary Table 4. Changes induced in the secretome of MAIT cells by IL-33 and IL-12p70 (Z-score).**

|  | **Control** | | | | **IL-33** | | | | **IL-12** | | | | **IL-33+IL-12** | | | |
| --- | --- | --- | --- | --- | --- | --- | --- | --- | --- | --- | --- | --- | --- | --- | --- | --- |
| **pg/mL** | **Donor 1** | **Donor 2** | **Donor 3** | **Donor 4** | **Donor 1** | **Donor 2** | **Donor 3** | **Donor 4** | **Donor 1** | **Donor 2** | **Donor 3** | **Donor 4** | **Donor 1** | **Donor 2** | **Donor 3** | **Donor 4** |
| **CCL11** | -0.436 | -0.484 | -0.306 | -0.422 | -0.307 | 2.977 | 0.654 | -0.533 | -0.393 | -0.309 | -0.653 | -0.449 | 2.031 | -0.489 | -0.473 | -0.407 |
| **CCL13** | -0.642 | -0.478 | -0.453 | 0.607 | -0.654 | 2.970 | 1.210 | 0.442 | -0.749 | -0.844 | -0.787 | -0.004 | 0.508 | -0.890 | -0.791 | 0.555 |
| **CCL19** | 0.094 | 0.524 | -0.828 | -1.238 | 0.176 | -0.398 | -0.431 | -1.180 | 1.913 | 1.930 | -0.112 | -1.027 | 0.896 | 1.158 | -0.880 | -0.598 |
| **CCL2** | -0.543 | -0.400 | -0.517 | 0.343 | -0.531 | -0.480 | -0.504 | 0.818 | -0.516 | -0.424 | -0.485 | 1.533 | -0.525 | -0.420 | -0.509 | 3.160 |
| **CCL3** | -0.021 | -1.134 | -0.965 | -0.585 | 0.603 | -0.981 | 0.241 | -0.128 | 0.530 | -1.295 | -0.926 | 0.240 | 2.039 | -0.473 | 0.827 | 2.025 |
| **CCL4** | -0.055 | -0.901 | -0.621 | 0.692 | 0.226 | -0.951 | -0.502 | 0.919 | -0.108 | -1.053 | -0.619 | 1.336 | 0.183 | -0.903 | -0.457 | 2.815 |
| **CCL7** | -0.507 | -0.428 | -0.492 | 0.313 | -0.481 | -0.475 | -0.501 | 0.775 | -0.494 | -0.461 | -0.512 | 1.569 | -0.502 | -0.467 | -0.500 | 3.164 |
| **CCL8** | -0.459 | -0.278 | -0.449 | -0.142 | -0.435 | -0.395 | -0.430 | 0.133 | -0.435 | -0.329 | -0.448 | 1.399 | -0.442 | -0.314 | -0.438 | 3.464 |
| **CSF1** | 0.632 | -0.948 | 0.064 | 0.276 | 1.151 | -0.476 | -0.055 | 0.834 | 0.744 | -1.966 | -0.595 | -0.065 | -0.190 | -1.636 | 0.022 | 2.209 |
| **CSF2** | 1.023 | -1.124 | -0.107 | 1.117 | 1.737 | -0.756 | -0.167 | 2.079 | -0.573 | -1.429 | -0.202 | -0.821 | 0.357 | -1.131 | 0.014 | -0.016 |
| **CSF3** | -0.178 | -0.893 | 1.678 | -0.449 | -0.737 | -0.459 | 0.276 | 1.557 | -0.345 | -1.095 | -1.165 | 0.190 | 0.044 | -0.857 | 0.100 | 2.333 |
| **CXCL10** | 0.299 | 0.485 | -0.334 | -1.666 | 0.711 | -0.589 | 0.694 | -1.608 | 1.884 | 0.247 | -0.170 | -1.292 | 1.522 | 0.767 | -0.153 | -0.798 |
| **CXCL11** | 0.075 | 0.694 | -0.692 | -1.665 | 0.548 | 0.151 | 0.074 | -1.684 | 1.018 | 1.890 | 0.628 | -1.270 | 0.915 | 0.683 | -0.332 | -1.034 |
| **CXCL12** | -0.459 | 0.572 | 0.111 | -0.529 | -0.472 | 0.279 | -0.257 | -0.268 | -0.116 | -0.733 | 3.604 | -0.391 | 0.277 | -0.720 | -0.594 | -0.306 |
| **CXCL8** | -0.274 | -0.514 | -0.449 | 0.794 | -0.071 | -0.611 | -0.448 | 1.203 | -0.491 | -0.927 | -0.524 | 0.752 | -0.564 | -0.618 | -0.418 | 3.160 |
| **CXCL9** | -0.082 | -0.360 | -0.517 | -1.437 | 0.637 | -0.583 | -0.054 | -1.305 | 1.384 | -0.585 | 0.620 | -0.694 | 2.813 | -0.071 | 0.034 | 0.199 |
| **EGF** | -0.409 | -0.489 | -0.460 | 3.178 | -0.366 | 1.743 | 0.310 | -0.559 | -0.407 | -0.577 | -0.645 | -0.009 | 0.144 | -0.603 | -0.567 | -0.284 |
| **FLT3LG** | -0.002 | -0.631 | 1.542 | -0.348 | -0.344 | -0.625 | 1.663 | -0.666 | -0.072 | -1.196 | 1.806 | -1.247 | -0.419 | -1.156 | 1.147 | 0.549 |
| **HGF** | -0.394 | -0.232 | 0.133 | -0.811 | -0.673 | 0.667 | 1.694 | -0.798 | -0.353 | -0.155 | 3.043 | -0.685 | -0.246 | -0.584 | 0.050 | -0.655 |
| **IFNG** | -0.680 | -0.692 | -0.669 | -0.699 | -0.602 | -0.665 | -0.432 | -0.622 | -0.357 | -0.580 | 0.335 | -0.361 | 2.159 | 1.255 | 2.399 | 0.212 |
| **IL10** | -0.541 | -0.738 | -0.805 | -1.134 | -0.805 | -0.938 | 0.320 | -0.990 | 0.628 | 0.624 | 1.281 | 2.749 | -0.441 | -0.103 | 0.316 | 0.576 |
| **IL13** | 0.289 | -1.056 | 1.634 | -1.146 | 0.774 | 0.711 | 0.346 | -1.131 | 0.920 | -0.794 | -0.230 | -1.450 | 1.606 | 0.336 | 0.508 | -1.315 |
| **IL15** | -0.134 | 0.194 | -0.758 | -0.670 | -0.262 | -0.118 | -0.927 | -0.484 | 0.721 | -0.464 | -1.224 | 1.789 | 0.062 | 0.233 | -0.735 | 2.777 |
| **IL17A** | -0.615 | -0.551 | -0.502 | -0.610 | -1.107 | 0.592 | 0.149 | 0.816 | -0.558 | -0.081 | -1.330 | 1.948 | -0.689 | 0.293 | -0.195 | 2.440 |
| **IL17C** | 0.583 | -0.312 | -0.312 | -0.312 | -0.312 | 3.781 | -0.312 | -0.312 | -0.312 | -0.312 | -0.312 | -0.312 | -0.312 | -0.312 | -0.312 | -0.312 |
| **IL17F** | -0.768 | -0.719 | -0.587 | -0.183 | -0.822 | -0.632 | 0.400 | 0.569 | -0.718 | -0.679 | -0.882 | 1.579 | 0.181 | 0.089 | 0.238 | 2.935 |
| **IL18** | 0.531 | 0.086 | 0.038 | -0.749 | 0.375 | 2.784 | 1.556 | -0.846 | 0.034 | -0.013 | -1.775 | -0.428 | -0.144 | -0.474 | -0.662 | -0.313 |
| **IL1B** | -0.293 | -0.735 | -0.152 | 0.392 | -0.662 | -0.686 | -0.065 | 0.731 | -0.624 | -0.836 | -0.006 | 1.586 | -0.606 | -0.687 | -0.384 | 3.027 |
| **IL2** | -0.806 | 0.961 | 1.632 | -0.723 | -0.030 | -1.008 | -0.469 | 0.494 | -0.634 | -0.888 | 0.787 | -0.058 | -0.280 | 2.524 | -0.320 | -1.183 |
| **IL27** | -0.518 | 1.764 | 0.198 | -1.052 | 0.569 | 0.654 | -0.626 | -0.881 | -0.159 | -0.639 | 2.728 | -0.755 | 0.310 | -0.389 | -0.860 | -0.345 |
| **IL33** | -0.760 | -0.760 | -0.757 | -0.763 | 1.508 | 0.780 | 1.292 | -0.763 | -0.759 | -0.753 | -0.761 | -0.763 | 1.740 | 1.008 | 1.275 | -0.763 |
| **IL4** | 0.361 | -0.064 | 0.372 | -1.107 | -0.858 | 0.197 | 0.622 | -0.869 | -0.365 | -1.191 | 2.758 | -1.272 | -0.080 | 1.021 | 0.804 | -0.331 |
| **IL6** | -0.783 | -0.765 | 0.184 | -0.070 | -0.798 | -0.808 | 0.106 | 1.348 | -0.652 | -0.773 | -0.240 | 0.820 | -0.243 | -0.557 | 0.148 | 3.082 |
| **IL7** | 0.149 | 0.766 | 0.782 | -1.675 | 0.699 | 0.195 | 0.517 | -1.675 | 0.402 | 0.217 | 0.713 | -1.676 | 0.407 | 1.204 | 0.651 | -1.676 |
| **LTA** | -0.557 | -0.658 | -0.338 | -0.895 | -0.340 | -0.594 | 1.937 | -0.820 | -0.113 | -0.521 | 0.265 | -0.728 | 0.946 | 0.335 | 2.682 | -0.600 |
| **MMP1** | 2.170 | -0.556 | -0.262 | 0.149 | 1.708 | -0.521 | 0.540 | 0.872 | -0.075 | -2.011 | -0.462 | -0.541 | 0.258 | -1.251 | -0.490 | 0.473 |
| **MMP12** | 2.563 | 0.065 | -0.194 | -0.554 | 2.550 | 0.078 | -0.323 | -0.264 | -0.040 | -0.740 | -0.529 | -0.768 | -0.210 | -0.587 | -0.371 | -0.675 |
| **OLR1** | -0.406 | -0.053 | 0.579 | -0.612 | -0.657 | -0.027 | 0.753 | -0.809 | -0.290 | 0.078 | 3.420 | -0.775 | -0.609 | -0.478 | 0.433 | -0.545 |
| **OSM** | -0.373 | -1.025 | 0.027 | -1.546 | -0.007 | -1.286 | 1.389 | -1.120 | 1.156 | -0.743 | 1.049 | -0.284 | 0.747 | -0.627 | 1.125 | 1.516 |
| **TGFA** | -0.208 | -0.501 | 1.092 | -0.749 | 0.056 | -0.419 | 2.330 | -0.628 | -0.326 | -0.714 | -1.240 | -0.629 | 0.036 | -0.379 | 2.293 | -0.012 |
| **TNF** | -0.428 | -1.286 | -0.971 | -0.670 | 0.187 | -1.007 | 0.313 | -0.391 | 0.200 | -1.176 | -0.598 | 0.174 | 2.104 | 0.387 | 1.386 | 1.777 |
| **TNFSF10** | 0.444 | 2.092 | -0.142 | -1.284 | -0.566 | 1.320 | 0.877 | -1.271 | 0.203 | -0.312 | 1.671 | -1.089 | -0.241 | -0.573 | -0.270 | -0.858 |
| **TNFSF12** | -0.048 | -0.465 | 0.048 | -0.651 | -0.203 | -0.044 | 0.513 | -0.610 | 0.134 | -0.543 | 3.655 | -0.622 | -0.163 | -0.636 | 0.065 | -0.429 |
| **TSLP** | -0.283 | -0.283 | -0.192 | -0.283 | -0.199 | -0.268 | -0.283 | -0.283 | -0.277 | -0.246 | 3.871 | -0.283 | -0.237 | -0.227 | -0.245 | -0.283 |
| **VEGFA** | -0.721 | -0.826 | -0.425 | -1.028 | -0.624 | -0.355 | 1.011 | -1.000 | 0.252 | -0.612 | 1.136 | -0.758 | 1.320 | 0.595 | 2.526 | -0.491 |
